# Put Yourself out There! A Strategy for Effective Self-Promotion in Academic Medicine

**DOI:** 10.15766/mep_2374-8265.11409

**Published:** 2024-06-18

**Authors:** Adam D. Wolfe, Lydia K. Davidson, Caroline R. Paul

**Affiliations:** 1 Associate Professor, Department of Pediatrics, Residency Program Director, and Assistant Dean of Education, Baylor College of Medicine and CHRISTUS Children's in San Antonio; 2 Fellow, Pediatric Hospital Medicine, Texas Tech University Health Sciences Center School of Medicine; 3 Associate Professor, Department of Pediatrics, New York University Grossman School of Medicine, NYU Langone Health

**Keywords:** Journaling, Peer Feedback, Problem-Action-Result, Self-Promotion, Communication Skills, Faculty Development, Feedback, Mentoring/Coaching, Promotions & Tenure

## Abstract

**Introduction:**

Trainees and faculty in academic medicine often struggle with self-promotion. Barriers may be more formidable for women and other groups underrepresented in medicine. Experience-based stories illustrating personal strengths are preferable when engaging in self-promotion activities.

**Methods:**

We developed a 90- to 120-minute workshop utilizing approaches such as iterative journaling and peer discussion to teach the development of problem-action-result (PAR) stories for self-promotion efforts in interviews and written applications to new positions. Participants provided Likert-scale (1 = *strongly disagree*, 5 = *strongly agree*) and free-response evaluations, which we analyzed for workshop strengths and areas for improvement.

**Results:**

We presented the workshop in person to 28 pediatric residents and subsequently to 22 residents, fellows, and faculty at an in-person national meeting. Sixty-one percent of the resident group and 100% of the national workshop group completed the evaluation. Both groups reported high satisfaction with the workshop's format (*M* = 4.7) and content (*M* = 4.7) and indicated intention to use the skills learned (*M* = 4.7). Strengths included the PAR format, interactivity, journaling, opportunity for reflection, and tips for interviewing and writing. Areas to improve included offering the workshop earlier in the academic year and providing more written examples of PAR stories.

**Discussion:**

This workshop used strategies of personal reflection, journaling, and peer feedback to help participants understand behavior-based recruiting practices and the PAR framework as a strategy for successful self-promotion. Learners can use these strategies to develop greater confidence and efficacy and to address barriers to effective self-promotion they encounter.

## Educational Objectives

By the end of this activity, learners will be able to:

1. Describe personal goals and strengths that inform their career path in medicine.
2. Employ PAR (problem-action-result) stories in writing personal statements and during interviews.
3. Develop strategies to overcome barriers to effective self-promotion.

## Introduction

Preparing for applications and interviews for a faculty, fellowship, or residency position in academic medicine can be daunting. These preparations, part of the core currency of recruitment and promotion practices, often challenge trainees and practicing academic physicians who are seeking the next phase of their career.^1–3^ We define *self-promotion* as the act of effectively articulating one's own accomplishments—verbally and in writing—for the purpose of recognition and advancement to a new position or role.^4^

An oft-reported barrier to active self-promotion is a perceived need for excessive humility and the concern that one's self-promotion efforts will be seen as bragging and therefore make one less warm or less likeable.^4^ Women in academic medicine face this perception to a greater degree than men, and there has been a call for improved mentorship and teaching strategies for self-promotion to address this challenge.^5–7^

Women experience other specific self-promotion barriers, including isolation, imposter syndrome, and perceived lack of negotiation skills. They also face common, pervasive myths about women in advancement processes, including that women are not interested in leadership roles, that they are not natural leaders, that they tend to opt out of careers, and even that we live in a post-sexist world.^8,9^ The workforce across nonmedical and medical domains remains inequitable on a gendered basis.^10–13^ Leadership at the table influences, and can therefore stymie, women's efforts at self-promotion. For example, women in academic medical careers report inconsistent, unclear, and sometimes capricious standards for promotion and tenure for women compared with male counterparts at their institutions.^14^ Furthermore, women physicians are often minoritized in leadership roles such as dean-level positions at allopathic US medical schools.^15^

While there has been less published about self-promotion barriers among underrepresented in medicine (URiM) academic physicians,^16^ evidence from the fields of business,^17^ law,^18^ and graduate education suggests similar perception gaps for self-promoting URiM individuals.^19^

The Association of American Medical Colleges encourages behavior-based interviewing for educational programs^20^ and instructs students to utilize their own experiences through stories when describing their strengths.^21^ These recommendations are based on the principle that past behavior predicts future behavior; stories told in self-promotion best illustrate genuine personal characteristics that may be attractive to evaluators. Graduate medical education programs successfully employ this approach in their recruiting efforts as well.^22^

A memorable strategy for briefly and effectively elaborating an experience that illustrates a personal strength is the problem-action-result (PAR) story.^23^ A PAR story, which can be written in a statement or cover letter or shared verbally during an interview, identifies a problem, challenge, or opportunity the individual has faced, describes the action the individual took to address it, and highlights the impact the action had on either the individual's own development or on others. This is an approach that is also routinely recommended by professional networks, such as Indeed,^24^ LinkedIn,^25^ Interview Genie,^26^ and others. A search of *MedEdPORTAL* and PubMed for the terms *self-promotion, problem-action-result,* and *PAR* revealed no teaching resources on this topic to date.

Our historical practice in educating and helping our residents apply to fellowships and faculty positions was based on individual mentorship meetings. It was not embedded in the curriculum. Following a review of the literature and local needs assessments from residents, fellows, and junior faculty, we developed a briskly paced, interactive workshop to teach the PAR strategy for showcasing core strengths in behavior-based interviews and in written statements using prior experience to demonstrate one's abilities. Participants iteratively develop their own lists of goals, key strengths, PAR stories, and potential barriers for their next self-promotion effort. Through iterative journaling activities and peer-facilitated feedback, participants develop and refine an outline of their next personal statement or cover letter, with additional focus on how mentoring and coaching can be optimized to address anticipated barriers to self-promotion.

## Methods

### Participants

We presented the workshop in person to pediatric residents and medical students during a residency program didactic session in San Antonio, TX, on July 22, 2022, and to a broader group of participants that included students, fellows, and teaching faculty at the Pediatric Academic Societies (PAS) annual meeting in Washington, DC, on April 30, 2023.

### Equipment and Room Needs

In each setting, the minimum required resources included room setup with round tables for small-group (four to five participants) discussion, a computer, and a projection system. Additional resources included live polling through Poll Everywhere and small blank journals distributed to learners. (We found many brands of mini-notebooks available through online retailers.) See the Discussion section for suggested alternatives to these resources.

### Duration of Workshops

The workshop was conducted once as a 120-minute experience and once as a 90-minute experience. The agenda in Appendix A provides timings for activities for both workshop lengths.

### Workshop Presentation

We used the agenda and instructions as described in Appendix A for facilitation of both workshops and presented the slides from Appendix B with speaker notes as shown with each slide. The workshop began with an introduction and review of objectives, and we distributed blank journals to all participants. We then used live polling (Poll Everywhere) to present the learner activation questions from Appendix C and gather information about the participants and their starting perceptions.

The first brief didactic focused on the workshop's purpose and an introduction of the gaps and barriers that may face women and members of URiM groups in academic medicine. We asked learners to list in their journal the most important qualities for the next position to which they would apply, to help set goals for the application; they then shared this information with colleagues in small groups of four to five. The next brief interactive didactic introduced behavior-based interviewing, with a discussion of the participants’ answers to learner activation question 6 (Appendix C) to evaluate which common interview questions were truly behavior based. The learners then used the journals to brainstorm their personal strengths, which would inform how they prepared for interviews and written statements about themselves. In small groups, they discussed these strengths and augmented their lists as appropriate.

We then introduced the PAR framework to the learners, using a sample interview question to illustrate how the response could be optimized to showcase the applicant's personal strength through a story. In their journal, residents drafted their first PAR story using an experience that illustrated one of the strengths they had previously identified, followed by a pair-share exercise to share and refine their story. We then moved into discussion of how to incorporate the PAR stories into interviews, personal statements, and cover letters. We shared a sample letter and asked small groups to identify examples of PAR stories within it (Appendix D, the sample letter on pp. 1–2 and the sample fellowship personal statement on p. 3 were handouts; pp. 4–6 were annotated for the facilitator), followed by a large-group discussion of the PAR examples in the letter. We then gave learners time to outline their next personal statement or cover letter in their journal, emphasizing how they could introduce their letter, which PAR stories they could use to illustrate selected strengths, and how to conclude the letter. They shared their outlines in small groups.

The final journal exercise was to outline a list of needs and barriers that they foresaw for their future self-promotion. In small groups, they shared their barriers and discussed strategies to address them. We also asked learners to revisit how their values, gender, ethnicity, race, LGBTQIA+ identity, or other aspects of their identity might affect how their self-promotion efforts could be viewed. We discussed the barriers together as a large group. For a wrap-up, we encouraged learners to continue to work on the five topics they had started journaling during this workshop. We distributed the evaluation (Appendix E) either electronically (using a Google Form during the resident workshop) or on paper (during the PAS workshop presentation) and encouraged completion before the learners left the workshop.

### Data Collection and Evaluation

We aggregated the responses to the live poll questions (Appendix C) after each workshop to summarize the composition of the learners and their reactions to interviewing and writing in self-promotion. Workshop evaluations (Appendix E) were gathered without identifying information and aggregated with descriptive statistics of scores on a 5-point Likert scale (1 = *strongly disagree,* 5 = *strongly agree*). We performed a topic analysis of the narrative comments, where we reviewed and categorized them into workshop strengths and areas for improvement, counting the frequency of specific categories raised in the evaluations. We assessed the impact of outcomes identified in the evaluations using the Kirkpatrick model.^27^

## Results

The resident workshop presentation in July 2022 had 28 participants, who during the live poll indicated training levels of PGY 3, PGY 2, PGY 1, and medical student. The PAS workshop presentation in April 2023 had 22 participants, who identified professional roles including resident, fellow, teaching faculty, educational leader, and research leader. All of the participants (100%) at both sessions participated in the learner activation poll during the workshop.

The Poll Everywhere learner activation responses to the word cloud questions (Appendix C, items 2 and 4) are shown in the Figure. Resident workshop participants indicated substantial anxiety, uncertainty, and apprehension about approaching both writing personal statements and cover letters (panel A) and interviewing (panel B). The PAS workshop audience exhibited some more-positive sentiments in their responses, noting excitement and confidence in their writing (panel C) and particularly in their interviewing (panel D) preparations, but many responses still skewed toward apprehension, uncertainty, and discomfort. These responses reinforced a shared sense of need among participants as they proceeded into the substance of the workshop.

**Figure.**
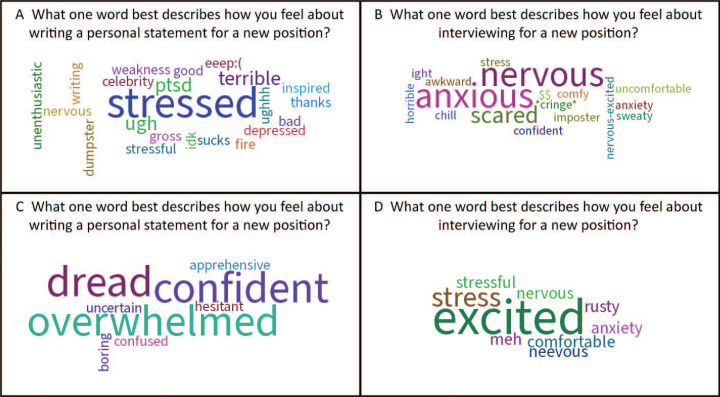
Word cloud responses from participants to the questions described. Panels A and B are responses from the resident workshop presentation (July 2022); panels C and D are responses from the Pediatric Academic Societies workshop presentation (April 2023).

At the July 2022 resident workshop, 61% of participants (17 of 28) completed the session evaluation (Appendix E); at the April 2023 PAS workshop, 100% of participants (22 of 22) completed it. Likert-scale responses from both workshops are summarized in Table 1. Both groups rated the workshop favorably, with mean responses of 4.6–4.9 among all questions. Participants from both groups identified the workshop content as useful (resident workshop *M* = 4.7) and relevant (PAS workshop *M* = 4.6). Participants in both groups indicated a high degree of intention to apply the skills practiced during the workshop (*M* = 4.7 in both groups).

**Table 1.**
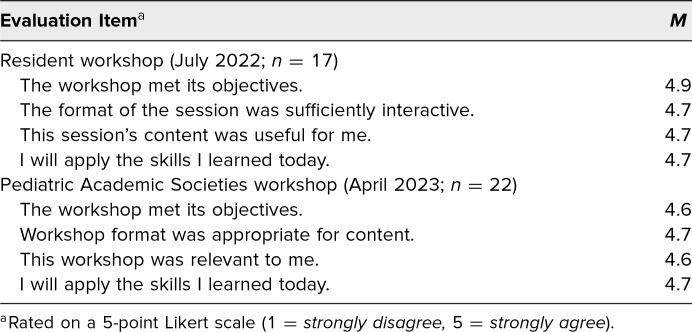
Summary of Postworkshop Evaluation Likert-Scale Responses

In the narrative portions of the evaluation (Appendix E), we counted the frequency of comments made in reference to strengths of the workshop (responses to items 6 and 8) and opportunities to improve the workshop (responses to items 7 and 9). The categories of strengths and areas for improvement are shown in Table 2. Most frequently noted strengths of the workshop included the PAR framework, tips for writing and interviewing, opportunity for reflection, and opportunity to interact with other participants. Opportunities to improve included offering the workshop to residents earlier in the academic year to help with fellowship application preparation, offering more time, sharing more examples of written statements, and holding more discussion about overcoming barriers.

**Table 2.**
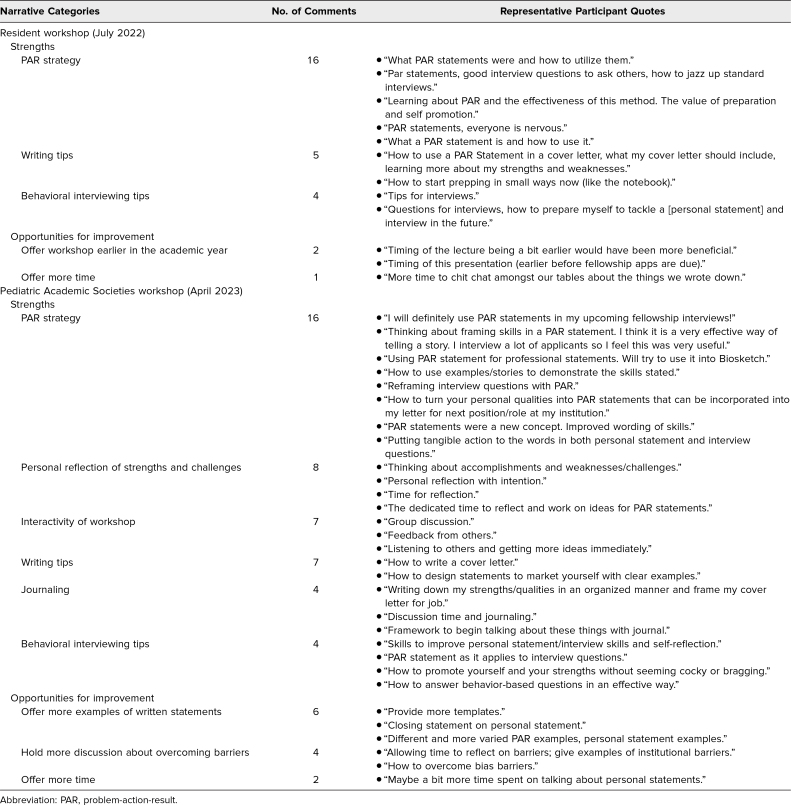
Summary of Postworkshop Evaluation Narrative Responses

### Postparticipation Feedback

Following the resident workshop in 2022, two residents notified the facilitators that they had found the content helpful in their own fellowship interviewing practices that summer. Following the PAS workshop, one fellow participant emailed us asking for feedback on a job application cover letter following the workshop's suggested strategy, and one of the faculty participants emailed to request guidance on using the workshop's tools to craft letters of recommendation.

## Discussion

The literature is lacking in effective training resources to prepare learners and faculty who coach learners with regard to self-promotion. Medical training programs increasingly employ behavior-based interviewing, which relies on personal stories to illustrate an applicant's strengths.^20–22^ In nonmedical recruitment practices, the PAR framework is routinely recommended to help applicants succeed in their applications.^23–26^

Feedback from our trainees revealed that they felt underprepared to write and speak about themselves as they applied to fellowships and job opportunities; thus, we initially developed this highly dynamic resource to help our residents locally. Subsequently, we adapted it for participants on a national level, including for teaching faculty. Historically, our advice to residents applying to jobs and fellowships has included an exploration of programs’ strategic mission, vision, and values in order to identify areas where the residents align with the programs. This is noted briefly during the slides in Appendix B. We usually conduct a separate workshop with trainees on recognizing and defining their personal strategic plans; that workshop is based on published recommendations available to educators.^28^

Using PAR stories, we introduced and employed an experience-based self-promotion strategy that is well established in many nonmedical fields, flexible to different applications, and memorable to learners. Via the implementation of the two workshops, we demonstrated that key ideas appearing first and known to be successful in nonmedical domains were indeed applicable to medical education, as demonstrated by the participants’ evaluations and experiences. It is important to consider resources and concepts described in the nonmedical literature to generate and expand domains that impact learners in medical education.

Our learners consisted of residents, fellows, and faculty across one local and one national workshop audience. Evaluation results confirmed learner satisfaction (Kirkpatrick level 1) and intent to use learned skills (Kirkpatrick level 2). Participants substantially valued the PAR framework and also appreciated the opportunities we afforded for self-reflection, interactivity, and journaling, as well as tips for interviewing and writing in their self-promotion efforts. The use of a small physical journal during the workshop was intended to provide participants with a list of goals, strengths, and PAR stories they could continue to refine following the workshop experience.

We saw a difference in response rates between the two presentations. At the resident workshop, only 17 of 28 participants completed the session evaluation. This evaluation was provided as an online form through a free Google Forms account. All 22 participants at the PAS workshop completed the evaluation, which was provided on paper. While we cannot establish the reason for the difference in response rates, we surmise that the resident participants may have found it easier to leave the session without following the link to the online form, while the PAS participants had ready access to the paper document on the table in front of them to facilitate completion.

The evaluations identified some areas for improvement. First, per the resident workshop, the timing of the workshop's implementation needed to be reconsidered. It was initially conducted in the latter part of July, after residents applying to fellowships had already submitted their personal statements. Narrative evaluation comments indicated that a presentation earlier in the academic year would be more helpful. Thus, we have offered subsequent iterations of the workshop in the late spring or early summer period to aid applicants in their fellowship application process. Second, regarding the PAS workshop, several participants requested more examples of PAR in written documents. This area of improvement was also echoed in a follow-up email from a participant seeking examples of letters of recommendation in which the writer used a PAR strategy to present the subject's strengths. Thus, for future implementation of the workshop, we are curating additional examples of actual and mock statements and letters that could be more helpful to faculty participants.

The workshop required some resources that may not be readily available to all users. We used Poll Everywhere, which has low-cost and free account options for online polling. There are other online tools that can also accommodate live group polling, if desired. Alternatively, if polling software is not used, the learner activation questions (Appendix C) could be shared verbally with audiences and answered by show of hands or by group brainstorming. We also purchased mini-notebooks for our participants. The mini-notebooks had a cost of approximately $20 per 48 notebooks and were procured through an online retailer. Other users could provide different journals, use plain paper, or simply ask participants to take notes on an electronic device during the journaling sections of the workshop. We feel that some form of active, dynamic writing makes the workshop more impactful, as the kinesthetic activity seems to help participants feel engaged and productive. For educators whose learners are not available together for in-person learning, the workshop could be facilitated without significant modification via a virtual meeting platform. This would require that each participant be able to access the writing materials and that the small-group activities be facilitated through the meeting platform by the facilitators. We would recommend against the session being recorded.

We recognize, as we continue our work, how the evaluations from our own workshop participants can impact future iterations of the workshop at local and national venues, with specific regard to women and members of URiM groups. Also, we continue to reflect upon the positions, biases, and lenses that we bring to the workshop in order to impact future iterations of it and other related educational resources at both the local and national levels. Indeed, two of the authors identify as women physicians with unique insights, with one having completed her chief residency and the other being a senior faculty member. Next steps include seeking more insight from participants, specifically women and members of URiM groups, to strengthen our understanding of barriers to self-promotion and inform future improvements to this resource.

An overall limitation is that we do not have systematic data regarding learner behavior changes after completion of the workshop (Kirkpatrick level 3). Some participants did contact us after each workshop to indicate that they were applying the learned workshop content to their application processes. Although lacking this learner outcome data, we have demonstrated the feasibility of an innovative career-planning medical education workshop that is generalizable to a wider group of learners as well as to teaching faculty who coach learners. Next steps include gauging learner usage and assessment of the skills taught during the workshop months to years following exposure to the content. Specifically, further research efforts could track fellows and those who have placed in their first jobs as well as examining the impact of the workshop on their application efforts.

In conclusion, we have developed and evaluated a workshop providing current and future academic physicians with a greater understanding of the philosophy behind behavior-based recruiting and the PAR strategy to allow them to harness their own experience to prove their strengths in writing and verbal encounters. We anticipate that these memorable and effective tools will allow learners or applicants to address their own barriers to self-promotion and have greater success in finding their next career position.

## Appendices


Facilitator Agenda.docxPut Yourself Out There.pptxPoll Questions.docxSample Letters.docxSession Evaluation.docx
*All appendices are peer reviewed as integral parts of the Original Publication.*
