# Supplementary material for: Put Yourself out There! A Strategy for Effective Self-Promotion in Academic Medicine
Source: MedEdPORTAL. 2024 Jun 18;20:11409. doi: 10.15766/mep_2374-8265.11409 (PMC11219085; doi:10.15766/mep_2374-8265.11409)
Supplement: Supplementary file 1 — Facilitator Agenda.docxPut Yourself Out There.pptxPoll Questions.docxSample Letters.docxSession Evaluation.docx [file mep_2374-8265.11409-s001.zip › C. Poll Questions.docx]

**Note to facilitator:** We implemented this introductory poll using Poll Everywhere ([www.polleverywhere.com](http://www.polleverywhere.com)). Each prompt is followed by the response format we chose within that platform. Other on-line survey tools could be used and the response formats adapted to what is available to you, or these questions could be answered without online tools using show of hands and discussion with your learner group.

1. Which best describes your current professional role?

Format: Multiple choice (revise options based on planned audience)

A. Medical Student

B. Resident

C. Teaching Faculty

D. Education Program Leader (e.g., Dean, GME leader, etc.)

E. Advanced Practice Professional

F. Other

2. What one word best describes how you feel about writing a personal statement for a new position?

Format: Word cloud

3. How comfortable are you with your skills at writing a personal statement?

Format: Likert scale/multiple choice

A. Very uncomfortable

B. Uncomfortable

C. Neither comfortable nor uncomfortable

D. Comfortable

E. Very comfortable

4. What one word best describes how you feel about interviewing for a new position?

Format: Word cloud

5. How comfortable are you with your skills at interviewing?

Format: Likert scale/multiple choice

A. Very uncomfortable

B. Uncomfortable

C. Neither comfortable nor uncomfortable

D. Comfortable

E. Very comfortable

6. What is the most common interview question you are used to hearing?

Format: Free response (using upvoting tool, if offered)
