# Supplementary material for: Put Yourself out There! A Strategy for Effective Self-Promotion in Academic Medicine
Source: MedEdPORTAL. 2024 Jun 18;20:11409. doi: 10.15766/mep_2374-8265.11409 (PMC11219085; doi:10.15766/mep_2374-8265.11409)
Supplement: Supplementary file 1 — Facilitator Agenda.docxPut Yourself Out There.pptxPoll Questions.docxSample Letters.docxSession Evaluation.docx [file mep_2374-8265.11409-s001.zip › D. Sample Letters.docx]

**Sample Letter #1 – Cover Letter for Leadership Position**

I am excited to apply for the position of Program Director of our Pediatrics Residency Training Program at our institution. It has been a privilege to serve as an Associate Program Director within the program for several years, to have built my career as a medical educator here, and to call this wonderful city mine and my family’s home.

As you know, my greatest passions lie in caring for patients and teaching learners. I have been teaching since 1998 and derive my greatest personal and professional satisfaction from helping people overcome challenges to succeed. This has driven my philosophy as an educator and leader over more than two decades: I feel successful as a physician, teacher, mentor, or leader when I can help others achieve their goals.

My work with our residency program began with creation of longitudinal curricula in resident scholarship and advanced communication skills. These curricula have led to national publications, invited presentations, and letters of thanks from our graduates who have gone on to practice the skills.

Following the unexpected departure of our Program Director a few years ago, I served for four months as acting Program Director until a new Program Director was appointed, and it was during this experience that I discovered my ambition to become the Program Director. It was immensely rewarding to have a more direct mentoring role, helping a struggling intern, and advocating for a resident who suffered professional mistreatment from a faculty physician. Over time each of these learners successfully reached resolutions to their challenges and made it to the next phases of their careers.

Since then, with direct mentorship, I have continued to seek opportunities to learn more about medical education leadership. I have authored our annual program improvement action plans, using resident and faculty feedback data. Each year I have led the efforts to develop our program improvement strategies, culminating last year with our first-ever leadership strategic planning retreat.

I also have directed this program’s intern recruitment process for the past four academic cycles. I have developed and refined a holistic scoring system for students applying to the program, a streamlined and inclusive interview day schedule, and a Rank List Committee that incorporates ten faculty members and resident input in our intern selection process. We had to make significant changes during the Covid-19 pandemic, and I have demonstrated that we can conduct virtual recruiting quite successfully in many ways. I deliberately diversified the pool of applicants that we interview and rank each year, with four consecutive years of increasingly diverse residents matching to this program to include those under-represented in medicine. I am proud to report that our current complement of trainees is the most representative of the community we serve compared with any prior year.

The emphasis on diversity, equity, and inclusion has also begun to permeate our curriculum over the past year. We began a series of workshops with our residents aimed at recognizing biases and microaggressions, and developing strategies to combat them. We also planned a quarterly DEI book club, but this was curtailed after one meeting due to the Covid virus surges; I intend to reintroduce this opportunity in the coming year.

I am particularly proud of this program’s ability to embrace curriculum changes in response to the needs of our learners and the evolving national landscape of Graduate Medical Education (GME) in pediatrics. I have been leading efforts to shepherd in two significant operational changes for this coming summer: the transition to a new rotation schedule, and the transition to a problem-based academic half day content curriculum. Both of these have required significant planning, meetings with stakeholders, and development of new evaluation strategies. I am confident that we are prepared to move into the future successfully, and that we are also well positioned to be flexible in the face of any unanticipated challenges that develop.

I have also accepted responsibility for working with all of our GME program leaders to develop and approve annual budgets, support ongoing and new program development, ensure accreditation compliance, mentor new faculty in leadership roles, and coordinate communication with medical school leadership. I serve as our site representative as a voting member of the Graduate Medical Education Executive Committee, the Dean’s Cabinet, and the College’s new strategic planning initiative.

It has been a true pleasure to network with medical education colleagues nationally. Over the past eight years, I have served in regional and national leadership roles in two organizations. This year I was also invited to serve as an Associate Editor of a top-tier, peer-reviewed medical education journal.

Reflecting on all of these experiences, I recognize that I have some more room to grow. My leadership approach is to be bold, innovative, collaborative, and unafraid of challenges. This leads me often to solve one problem and immediately seek out another to address. I wish to work more on pausing to celebrate victories and successes with my teams. I also am developing my skills in delegating responsibility. While the Program Director should be capable of performing all tasks in support of the program, there must also be a strong culture of trust and delegation of tasks.

Having spent eight years building a successful career in medical education with a focus on pediatric residency leadership, I am ready to take the next step to formally lead our residency program. It has been an honor to serve our exceptional learners so far, and we have much more we can accomplish as we build the region’s premier pediatrics program.

Thank you for considering my application, and please let me know if I can provide additional information.

**Sample Letter #2 – Personal Statement for Fellowship Application**

Making the decision to pursue more training is not made lightly. There is an argument that one can still go into practice of pediatric hospital medicine without the additional fellowship; however, I believe many of us feel an innate desire to continue to expand our knowledge and skill sets. I feel I strongly fall into the latter group due to my compulsion to continuously improve, my commitment to education, and my sheer

tenacity.

While in residency I served on a number of committees and found solutions to commonly encountered issues. I enjoyed being a part of the committee that regularly reviewed the residency program and another multidisciplinary board that analyzed different significant events. I was also able to create change on my own when it became apparent our patient population was having difficulty filling prescriptions upon discharge. I took the initiative to collaborate with the outpatient pharmacy to create a “meds to bed” program. I modified the way we were e-prescribing in order to communicate delivery to the bedside was needed. This changed the utilization of our internal resources and the culture of our discharge process. Optimizing this method of prescribing resulted in fewer bounce-back admissions due to medication unavailability and decreased the number of calls the residents received from the pharmacy or patient’s family upon discharge.

While in residency I also became well known for my joy of teaching. I often had medical students or interns with me to teach at the bedside, speak with the nurses to gain their point of view, or to go over a PowerPoint on a high-yield topic. One recent example came during my year as Chief Resident. Our program had difficulty scheduling a faculty member from Cardiology to present content at our academic half day regarding common pediatric EKG abnormalities and conduction problems. I developed a two-hour interactive session that blended clinical cases, brief didactics, and game-based board review to cover this complex and challenging topic with the residents. They evaluated my teaching very highly and found the session engaging. I have had this natural tendency to teach since being an undergraduate and would like to continue to share my fascination in medicine with learners as long as I am able.

Lastly, I have faced many setbacks and challenges but have not lost sight of my goal to practice pediatric hospital medicine. While in medical school, I discovered I had test-taking anxiety and lacked a number of test-taking skills; however, with time, I was able to overcome these and continue to pass required standardized examinations. I also learned that taking the more difficult path forces one to appreciate how badly they desire the end destination. My most recent challenge has been becoming a PGY-4 Chief Resident at a different program halfway across the country. This role became even more daunting when I discovered my co-Chief Resident could no longer accept her position for extenuating circumstances. Although it was a unique situation, I maintained my optimism and have already brought an outsider perspective to leadership, curriculum, and program evaluation in the first part of the academic year.

With all of this in mind, I believe I am well-suited for a fellowship and career in pediatric hospital medicine. I am open and interested in change, intellectually curious, and sure of my career goal. While in fellowship I hope to learn more about practicing quality improvement initiatives and using evidence-based medicine to advance best practices in pediatric hospital medicine. I would like to have more formal training in medical education and to continue to work with medical students and residents. Ultimately I would like to be a Pediatric Hospitalist at an academic center and to serve as an educator.

Thank you for taking the time to consider my application for your fellowship program.

**Sample Letter #1 – Cover Letter for Leadership Position (annotated)**

I am excited to apply for the position of Program Director of our Pediatrics Residency Training Program at our institution. It has been a privilege to serve as an Associate Program Director within the program for several years, to have built my career as a medical educator here, and to call this wonderful city mine and my family’s home.

As you know, my greatest passions lie in caring for patients and teaching learners. I have been teaching since 1998 and derive my greatest personal and professional satisfaction from helping people overcome challenges to succeed. This has driven my philosophy as an educator and leader over more than two decades: I feel successful as a physician, teacher, mentor, or leader when I can help others achieve their goals.

PAR

My work with our residency program began with creation of longitudinal curricula in resident scholarship and advanced communication skills. These curricula have led to national publications, invited presentations, and letters of thanks from our graduates who have gone on to practice the skills.

Following the unexpected departure of our Program Director a few years ago, I served for four months as acting Program Director until a new Program Director was appointed, and it was during this time that I discovered my ambition to become the Program Director. It was immensely rewarding to have a more direct mentoring role, helping a struggling intern, and advocating for a resident who suffered professional mistreatment from a faculty physician. Over time each of these learners successfully reached resolutions to their challenges and made it to the next phases of their careers.

PAR

Since then, with direct mentorship, I have continued to seek opportunities to learn more about medical education leadership. I have authored our annual program improvement action plans, using resident and faculty feedback data. Each year I have led the efforts to develop our program improvement strategies, culminating last year with our first-ever leadership strategic planning retreat.

I also have directed this program’s intern recruitment process for the past four academic cycles. I have developed and refined a holistic scoring system for students applying to the program, a streamlined and inclusive interview day schedule, and a Rank List Committee that incorporates ten faculty members and resident input in our intern selection process. We had to make significant changes during the Covid-19 pandemic, and I have demonstrated that we can conduct virtual recruiting quite successfully. I deliberately diversified the pool of applicants that we interview and rank each year, with four consecutive years of increasingly diverse residents matching to this program to include those under-represented in medicine. I am proud to report that our current complement of trainees is the most representative of the community we serve compared with any prior year.

PAR

The emphasis on diversity, equity, and inclusion has also begun to permeate our curriculum over the past year. We began a series of workshops with our residents aimed at recognizing biases and microaggressions, and developing strategies to combat them. We also planned a quarterly DEI book club, but this was curtailed after one meeting due to the Covid virus surges; I intend to reintroduce this opportunity in the coming year.

I am particularly proud of this program’s ability to embrace curriculum changes in response to the needs of our learners and the evolving national landscape of Graduate Medical Education (GME) in pediatrics. I have been leading efforts to shepherd in two significant operational changes for this coming summer: the transition to a new rotation schedule, and the transition to a problem-based academic half day content curriculum. Both of these have required significant planning, meetings with stakeholders, and development of new evaluation strategies. I am confident that we are prepared to move into the future successfully, and that we are also well positioned to be flexible in the face of any unanticipated challenges that develop.

PAR

I have also accepted responsibility for working with all of our GME program leaders to develop and approve annual budgets, support ongoing and new program development, ensure accreditation compliance, mentor new faculty in leadership roles, and coordinate communication with medical school leadership. I serve as our site representative as a voting member of the Graduate Medical Education Executive Committee, the Dean’s Cabinet, and the College’s new strategic planning initiative.

PAR

It has been a true pleasure to network with medical education colleagues nationally. Over the past eight years, I have served in regional and national leadership roles in two organizations. This year I was also invited to serve as an Associate Editor of a top-tier, peer-reviewed medical education journal.

Reflecting on all of these experiences, I recognize that I have some more work to do. My leadership approach is to be bold, innovative, collaborative, and unafraid of challenges. This leads me often to solve one problem and immediately seek out another to address. I wish to work more on pausing to celebrate victories and successes with my teams. I also am developing my skills in delegating responsibility. While the Program Director should be capable of performing all tasks in support of the program, there must also be a strong culture of trust and delegation of tasks.

Areas for growth

Having spent eight years building a successful career in medical education with a focus on pediatric residency leadership, I am ready to take the next step to formally lead our residency program. It has been an honor to serve our exceptional learners so far, and we have much more we can accomplish as we build the region’s premier pediatrics program.

Thank you for considering my application, and please let me know if I can provide additional information.

**Sample Letter #2 – Personal Statement for Fellowship Application (annotated)**

Making the decision to pursue more training is not made lightly. There is an argument that one can still go into practice of pediatric hospital medicine without the additional fellowship; however, I believe many of us feel an innate desire to continue to expand our knowledge and skill sets. I feel I strongly fall into the latter group due to my compulsion to continuously improve, my commitment to education, and my sheer

tenacity.

While in residency I served on a number of committees and found solutions to commonly encountered issues. I enjoyed being a part of the committee that regularly reviewed the residency program and another multidisciplinary board that analyzed different significant events. I was also able to create change on my own when it became apparent our patient population was having difficulty filling prescriptions upon discharge. I took the initiative to collaborate with the outpatient pharmacy to create a “meds to bed” program. I modified the way we were e-prescribing in order to communicate delivery to the bedside was needed. This changed the utilization of our internal resources and the culture of our discharge process. Optimizing this method of prescribing resulted in fewer bounce-back admissions due to medication unavailability and decreased the number of calls the residents received from the pharmacy or patient’s family upon discharge.

PAR

While in residency I also became well known for my joy of teaching. I often had medical students or interns with me to teach at the bedside, speak with the nurses to gain their point of view, or to go over a PowerPoint on a high-yield topic. One recent example came during my year as Chief Resident. Our program had difficulty scheduling a faculty member from Cardiology to present content at our academic half day regarding common pediatric EKG abnormalities and conduction problems. I developed a two-hour interactive session that blended clinical cases, brief didactics, and game-based board review to cover this complex and challenging topic with the residents. They evaluated my teaching very highly and found the session engaging. I have had this natural tendency to teach since being an undergraduate and would like to continue to share my fascination in medicine with learners as long as I am able.

PAR

Lastly, I have faced many setbacks and challenges but have not lost sight of my goal to practice pediatric hospital medicine. While in medical school, I discovered I had test-taking anxiety and lacked a number of test-taking skills; however, with time, I was able to overcome these and continue to pass required standardized examinations. I also learned that taking the more difficult path forces one to appreciate how badly they desire the end destination. My most recent challenge has been becoming a PGY-4 Chief Resident at a different program halfway across the country. This role became even more daunting when I discovered my co-Chief Resident could no longer accept her position for extenuating circumstances. Although it was a unique situation, I maintained my optimism and have already brought an outsider perspective to leadership, curriculum, and program evaluation in the first part of the academic year.

PAR

PAR

With all of this in mind, I believe I am well-suited for a fellowship and career in pediatric hospital medicine. I am open and interested in change, intellectually curious, and sure of my career goal. While in fellowship I hope to learn more about practicing quality improvement initiatives and using evidence-based medicine to advance best practices in pediatric hospital medicine. I would like to have more formal training in medical education and to continue to work with medical students and residents. Ultimately I would like to be a Pediatric Hospitalist at an academic center and to serve as an educator.

Areas for growth

Thank you for taking the time to consider my application for your fellowship program.
