# Supplementary material for: Put Yourself out There! A Strategy for Effective Self-Promotion in Academic Medicine
Source: MedEdPORTAL. 2024 Jun 18;20:11409. doi: 10.15766/mep_2374-8265.11409 (PMC11219085; doi:10.15766/mep_2374-8265.11409)
Supplement: Supplementary file 1 — Facilitator Agenda.docxPut Yourself Out There.pptxPoll Questions.docxSample Letters.docxSession Evaluation.docx [file mep_2374-8265.11409-s001.zip › E. Session Evaluation.docx]

1. This workshop content was relevant to me.

- Strongly disagree
- Disagree
- Neutral
- Agree
- Strongly agree

2. This workshop met its learning objectives.

- Strongly disagree
- Disagree
- Neutral
- Agree
- Strongly agree

3. The workshop facilitators were effective.

- Strongly disagree
- Disagree
- Neutral
- Agree
- Strongly agree

4. The workshop format was appropriate for its content.

- Strongly disagree
- Disagree
- Neutral
- Agree
- Strongly agree

5. I will apply the skills I learned today.

- Strongly disagree
- Disagree
- Neutral
- Agree
- Strongly agree

6. What did you learn today that you plan to apply to your professional development?

7. What is still unclear?

8. What was the most valuable aspect of this session?

9. How can we improve this session?
