# Supplementary material for: Put Yourself out There! A Strategy for Effective Self-Promotion in Academic Medicine
Source: MedEdPORTAL. 2024 Jun 18;20:11409. doi: 10.15766/mep_2374-8265.11409 (PMC11219085; doi:10.15766/mep_2374-8265.11409)
Supplement: Supplementary file 1 — Facilitator Agenda.docxPut Yourself Out There.pptxPoll Questions.docxSample Letters.docxSession Evaluation.docx [file mep_2374-8265.11409-s001.zip › A. Facilitator Agenda.docx]

**Put Yourself Out There! A Strategy for Effective Self-Promotion in Academic Medicine**

**Educational Objectives**

By the end of this activity, learners will be able to:

1. Describe personal goals and strengths that inform one’s career path in medicine
2. Employ PAR (Problem-Action-Result) stories in writing personal statements and during interviews
3. Develop strategies to overcome barriers to effective self-promotion

**Notes**

This workshop has been offered in both 90- and 120-minute formats. The longer format allows for more exploration of goals and small group discussion. The agenda below has timing columns for both workshop lengths. Customize the sections as needed depending on the participants. Within each section of the agenda below are notes for the facilitator, and notes for which slides in Appendix B and which handouts apply to each activity.

In the slide deck (Appendix B), each of the 5 journaling activities has a photo of stacked journals on the appropriate slide as an anchor.

**Agenda**

| Activity time based on total length (min) | |  | Slide # | Objectives |
| --- | --- | --- | --- | --- |
| 120’ total | 90’ total | Activity | (Appendix B) | Addressed |
| 5 | 5 | Introduction and review objectives   - Introduce facilitators and the objectives of the workshop. - Distribute the blank journals to all learners. | 1-2 | 1-3 |
| 10 | 10 | Live poll, learner activation   - Provide instructions for learners to access the live poll, using the questions provided in **Appendix C** (can be using electronic polling such as [www.Polleverywhere.com](http://www.Polleverywhere.com) or large group discussion). - Define the “next position” the learners might be seeking as a residency match (for students), a fellowship match (for residents), a new employment position, or a new appointment within the current institution. - Discuss responses to questions 2-5 with the group as needs assessment for the forthcoming content in the workshop. - Inform learners that the responses to question 6 will be discussed later in the workshop. | 3 | 1, 3 |
| 10 | 10 | Didactic – Purpose of workshop, definitions, identified gaps in self-promotion with regards to women and members of URiM groups   - Discuss the points on the slides and included in the slide speaker notes. - Emphasize the importance of recognizing that some people who actively self-promote may be disadvantaged by being viewed differently; this includes impact on women and members of URiM groups. - Raise awareness of this potential barrier for some professionals, inform learners that this topic will also be addressed again later in the workshop. | 4-6 | 1, 3 |
| 10 | - | Journal exercise #1 – What do I want?   - Instruct learners to list in the journal the most important qualities of the next position they will apply to. Sample qualities to consider are provided in the slide. - Ask them to go through their list and identify up to three of these qualities of the next position that were the most important to them. - Discuss their top needs for their next position in small groups of 4-5. - Encourage learners to augment their own lists if the discussion helped them identify new qualities to add. | 7-8 | 1 |
| 5 | 5 | Didactic – Introduction to behavior-based interviewing   - Define behavior-based interviewing. - Share participant responses from the learner activation (**Appendix C, question 6**) to critique in large group whether common interview questions conform to a behavior-based format or not. - Emphasize that behavior-based questions require a concrete, past tense response of a specific event or experience. Encourage learners to keep this principle in mind as they apply for their next position. | 9 | 1 |
| 15 | 10 | Journal exercise #2 – What makes me stand out?   - Instruct learners to list their personal strengths in the journal. Examples include intellectual curiosity, work ethic, leadership, heart for service, positivity, faith, communication skills, and teamwork. - Emphasize that this can be a challenging task and encourage them not to be humble about naming their strengths. - Asked them to identify three strengths that they consider their greatest strengths. - Ask learners to work in small groups of 4-5 and share the strengths they listed. - Encourage learners to augment their own lists if the discussion helps them identify new strengths to add. | 10-11 | 1 |
| 5 | 5 | Didactic – Introduction to PAR framework   - Introduce the PAR strategy for highlighting one’s strengths. The purpose of PAR is to ensure that prior behavior highlights skills or abilities that the applicant will exhibit in the next position as well. | 12-13 | 2 |
| 15 | 10 | Journal exercise #3 – Draft a PAR story   - Ask the learners to develop a brief PAR story from one of the personal strengths they listed earlier. If time permits, develop more than one PAR story. - Ask learners to work in pairs or trios and share a PAR story with their partner(s). The listener should provide feedback on the partner’s PAR story to help make it more effectively represent the partner’s chosen strength. | 14-15 | 2 |
| 10 | 10 | Didactic and discussion – How to incorporate PAR stories into interviews, personal statements, and cover letters   - Show tips for bringing PAR stories into a behavior-based and a non-behavior-based interview setting. In either case the strategy allows the applicant to showcase their chosen strengths. A similar approach can be used to emphasize one’s strengths in a personal statement or cover letter - Provide the sample cover letter (**Appendix D, pages 1-2 - handout**) and/or sample fellowship personal statement (**Appendix D, page 3 – handout**). In small groups of 4-5, ask learners to review the sample letter(s) and identify examples of PAR stories. - Identify examples of complete or partial PAR stories in this example letter in the large group (**Appendix D, page 4-5 and page 6 – annotated copies, highlighted in yellow**). | 16-17 | 2 |
| 15 | 10 | Journal exercise #4 – Outline a personal statement or cover letter   - Instruct learners to begin outlining their next cover letter or personal statement in the journal. The slide includes tips for information to include in the introductory and concluding paragraphs, and where to insert PAR stories. - Ask the learners to work in small groups of 4-5 and share their outlines. - Encourage them to consider how they might include areas for personal growth or improvement in their letter as well. Reference an illustration in the sample cover letter (**Appendix D, page 2, paragraph 4**) and/or sample fellowship personal statement (**Appendix D, page 3, next-to-last paragraph**). | 18-19 | 2 |
| 15 | 10 | Journal exercise #5 – What is in my way? Barriers/needs for self-promotion  Instruct learners to identify potential barriers to their successful self-promotion and outline them in the journal.  Aski them to work in small groups of 4-5 and share the barriers they listed and consider strategies to address them. Also revisit how one’s values, gender, ethnicity, race, LGBTQ+ identity, or other aspects of their identity might affect how their efforts at self-promotion could be viewed.  Open this discussion to the large group to discuss barriers together. Prompts for this discussion are included in the slide speaker notes. | 20-21 | 3 |
| 5 | 5 | Wrap up and evaluation   - Provide take home points, including encouraging learners to continue to work on the five topics they started journaling during this workshop. - Distribute the evaluation (**Appendix E**) either electronically (using a Google Form or other survey platform) or on paper and encourage completion before learners leave the workshop. | 22 | 1-3 |
