# Supplementary material for: Put Yourself out There! A Strategy for Effective Self-Promotion in Academic Medicine
Source: MedEdPORTAL. 2024 Jun 18;20:11409. doi: 10.15766/mep_2374-8265.11409 (PMC11219085; doi:10.15766/mep_2374-8265.11409)
Supplement: Supplementary file 1 — Facilitator Agenda.docxPut Yourself Out There.pptxPoll Questions.docxSample Letters.docxSession Evaluation.docx [file mep_2374-8265.11409-s001.zip › B. Put Yourself Out There.pptx]

## Slide 1
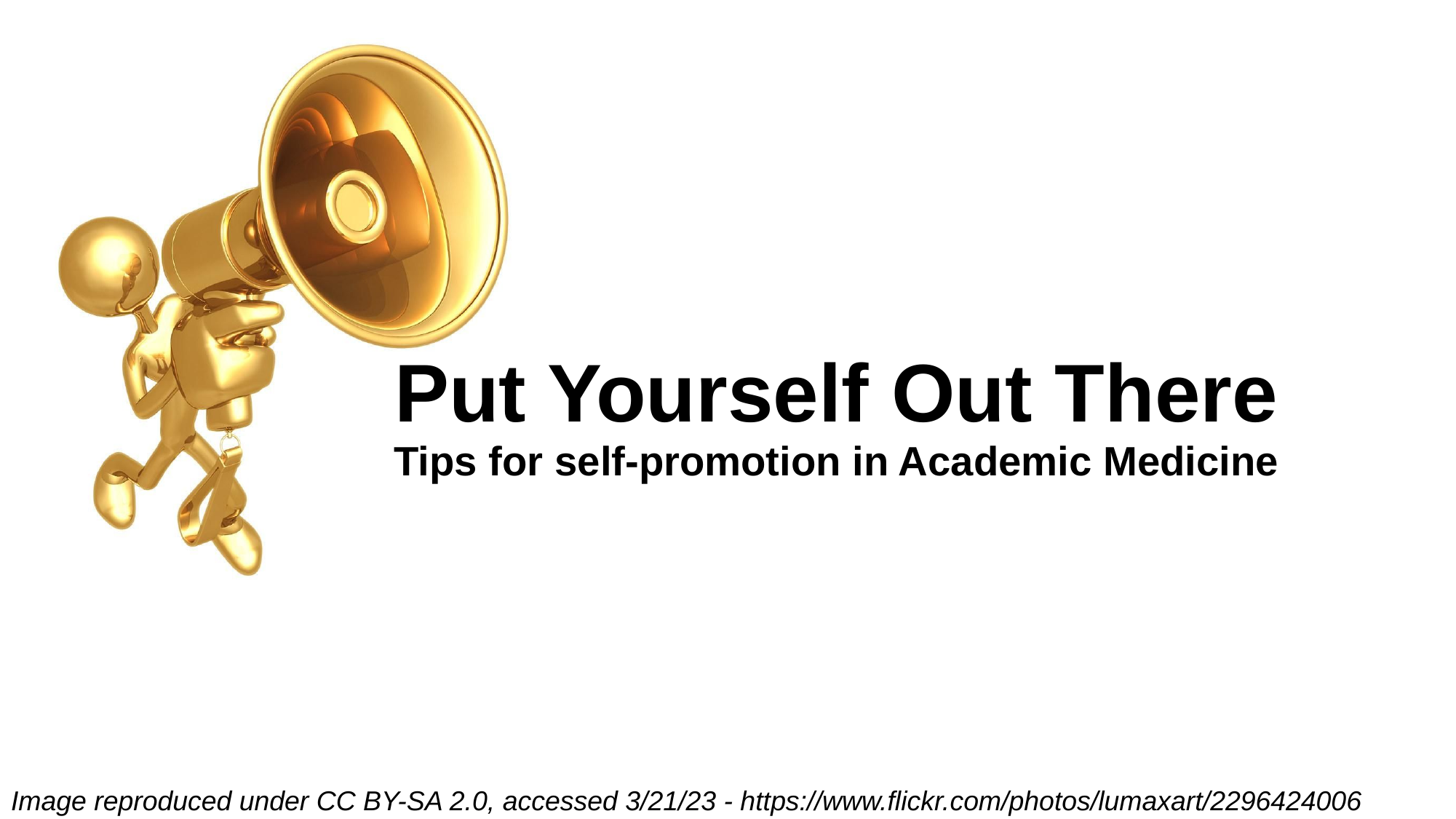

# Put Yourself Out There
Tips for self-promotion in Academic Medicine
Image reproduced under CC BY-SA 2.0, accessed 3/21/23 - https://www.flickr.com/photos/lumaxart/2296424006

## Slide 2
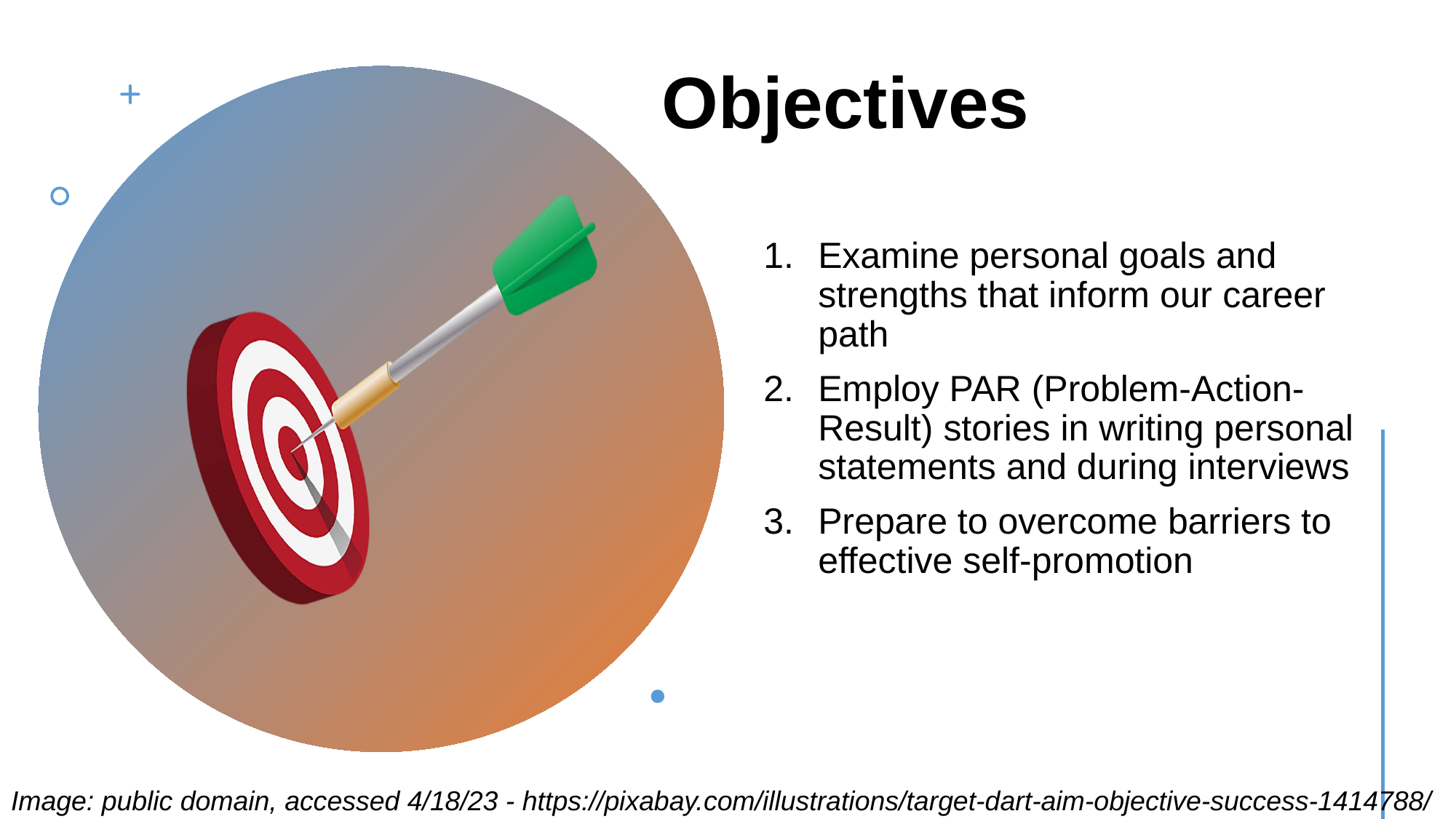

# Objectives
Examine personal goals and strengths that inform our career path
Employ PAR (Problem-Action-Result) stories in writing personal statements and during interviews
Prepare to overcome barriers to effective self-promotion
Image: public domain, accessed 4/18/23 - https://pixabay.com/illustrations/target-dart-aim-objective-success-1414788/

## Slide 3
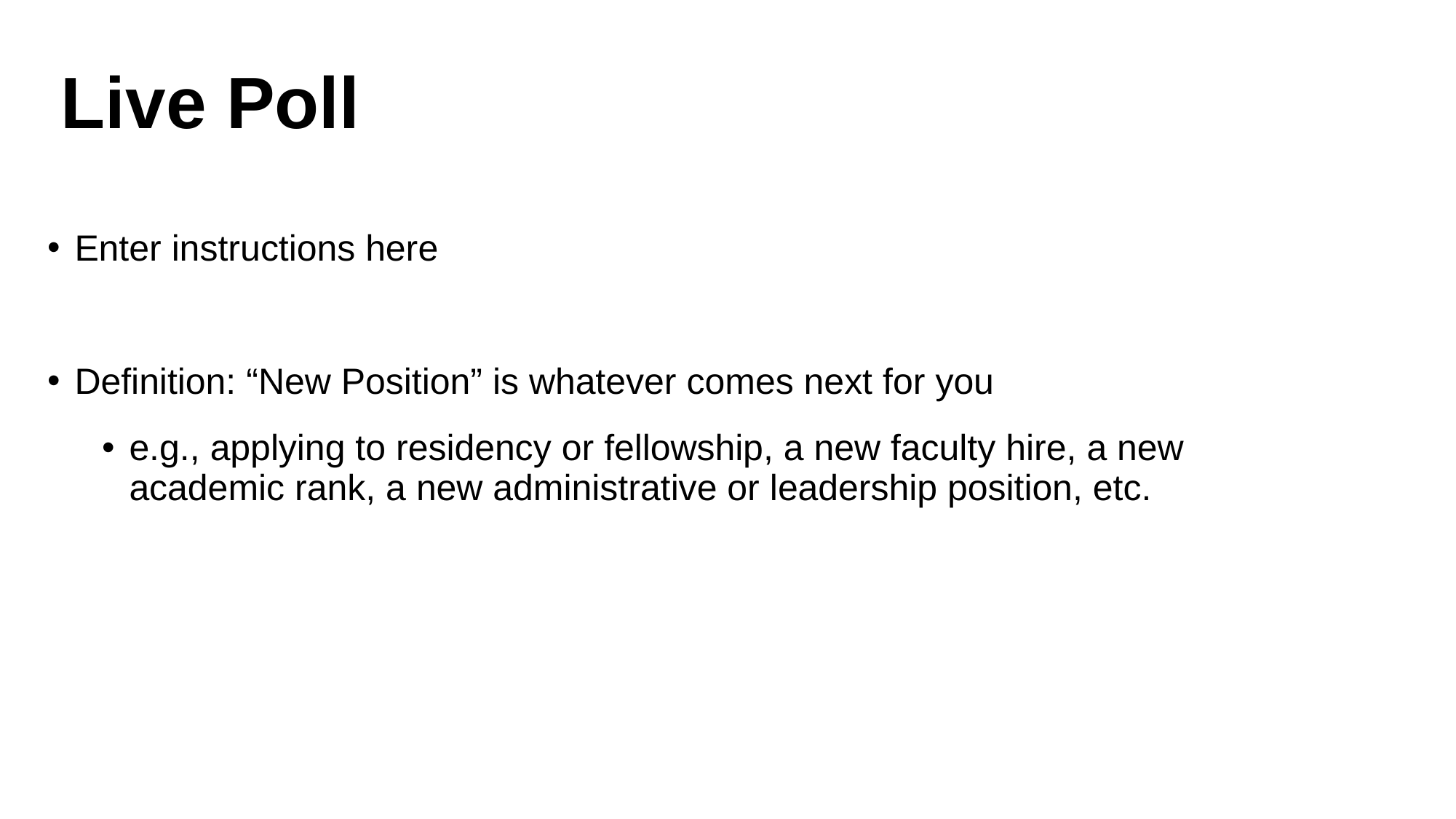

Live Poll
Enter instructions here
Definition: “New Position” is whatever comes next for you
e.g., applying to residency or fellowship, a new faculty hire, a new academic rank, a new administrative or leadership position, etc.

## Slide 4
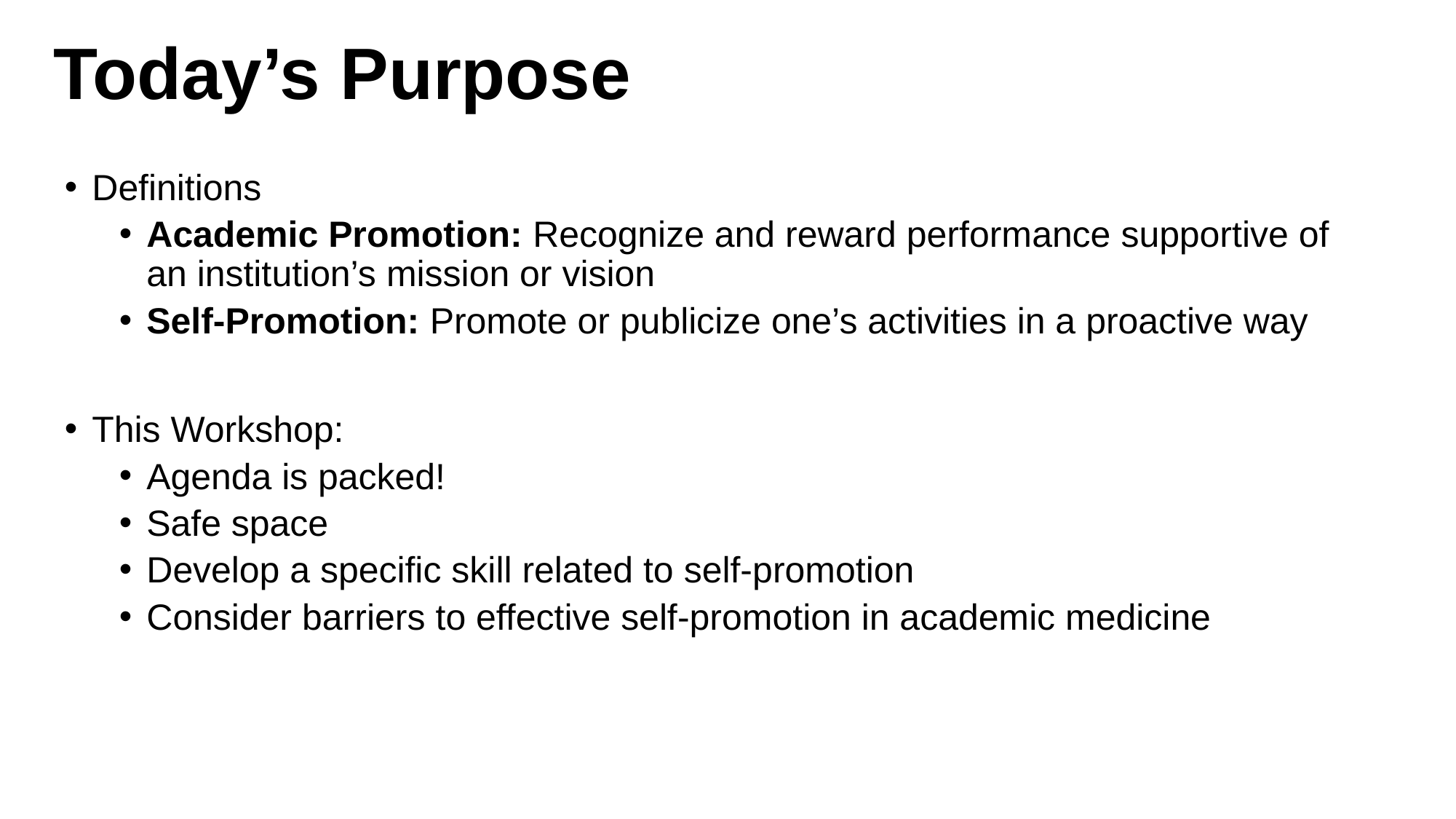

Today’s Purpose
Definitions
Academic Promotion: Recognize and reward performance supportive of an institution’s mission or vision
Self-Promotion: Promote or publicize one’s activities in a proactive way
This Workshop:
Agenda is packed!
Safe space
Develop a specific skill related to self-promotion
Consider barriers to effective self-promotion in academic medicine

## Slide 5
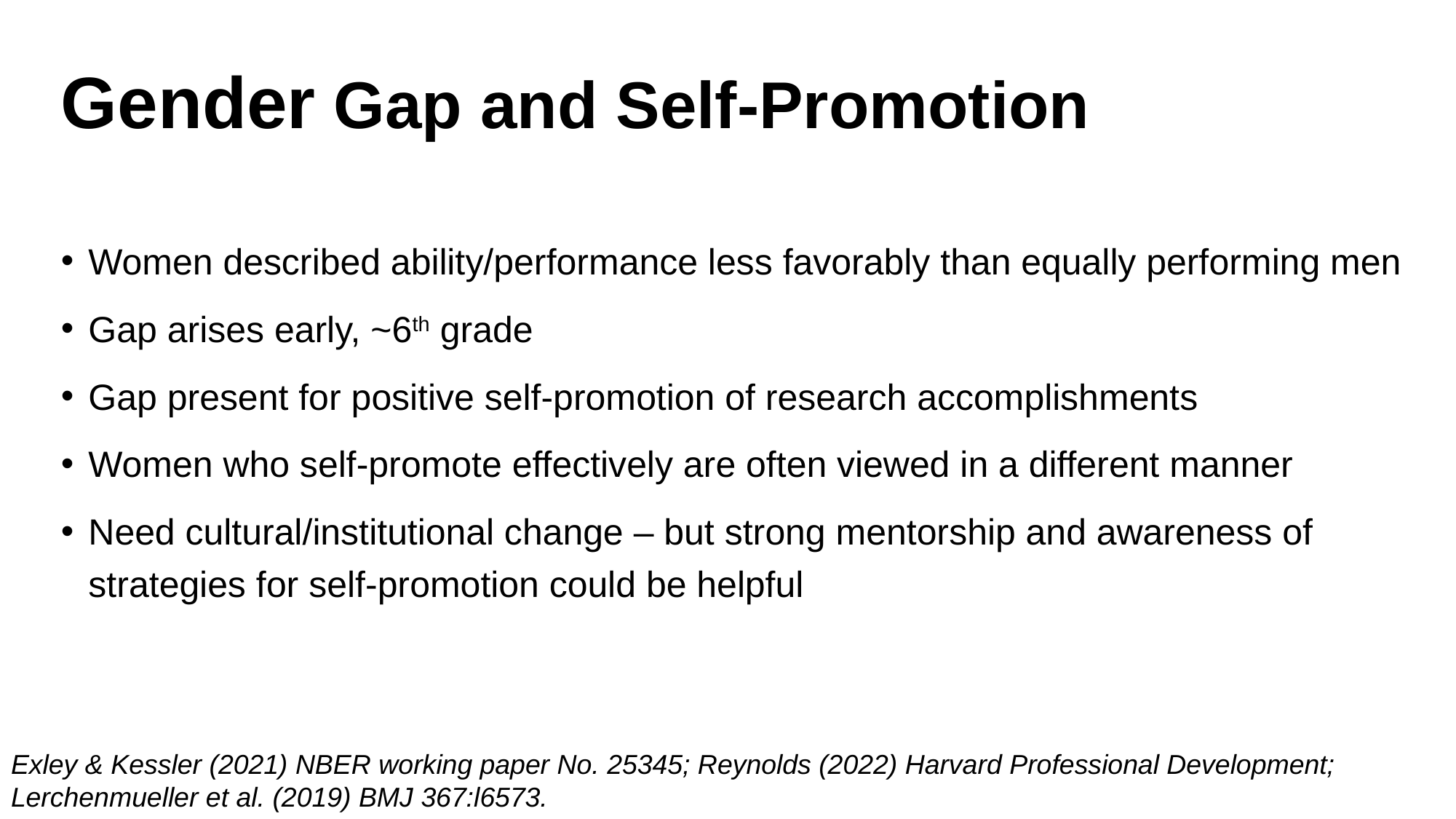

# Gender Gap and Self-Promotion
Women described ability/performance less favorably than equally performing men
Gap arises early, ~6th grade
Gap present for positive self-promotion of research accomplishments
Women who self-promote effectively are often viewed in a different manner
Need cultural/institutional change – but strong mentorship and awareness of strategies for self-promotion could be helpful
Exley & Kessler (2021) NBER working paper No. 25345; Reynolds (2022) Harvard Professional Development; Lerchenmueller et al. (2019) BMJ 367:l6573.

## Slide 6
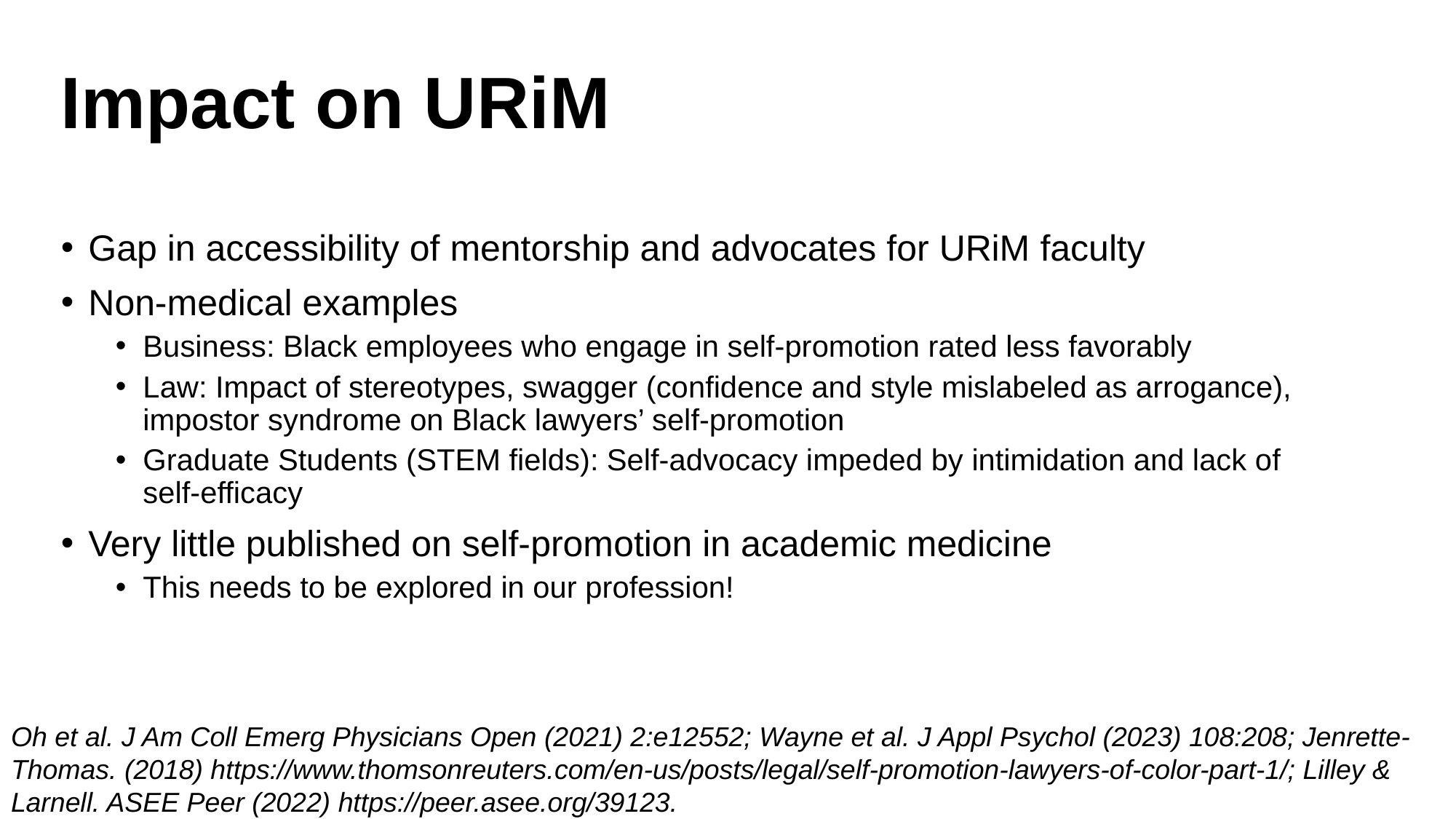

# Impact on URiM
Gap in accessibility of mentorship and advocates for URiM faculty
Non-medical examples
Business: Black employees who engage in self-promotion rated less favorably
Law: Impact of stereotypes, swagger (confidence and style mislabeled as arrogance), impostor syndrome on Black lawyers’ self-promotion
Graduate Students (STEM fields): Self-advocacy impeded by intimidation and lack of self-efficacy
Very little published on self-promotion in academic medicine
This needs to be explored in our profession!
Oh et al. J Am Coll Emerg Physicians Open (2021) 2:e12552; Wayne et al. J Appl Psychol (2023) 108:208; Jenrette-Thomas. (2018) https://www.thomsonreuters.com/en-us/posts/legal/self-promotion-lawyers-of-color-part-1/; Lilley & Larnell. ASEE Peer (2022) https://peer.asee.org/39123.

## Slide 7
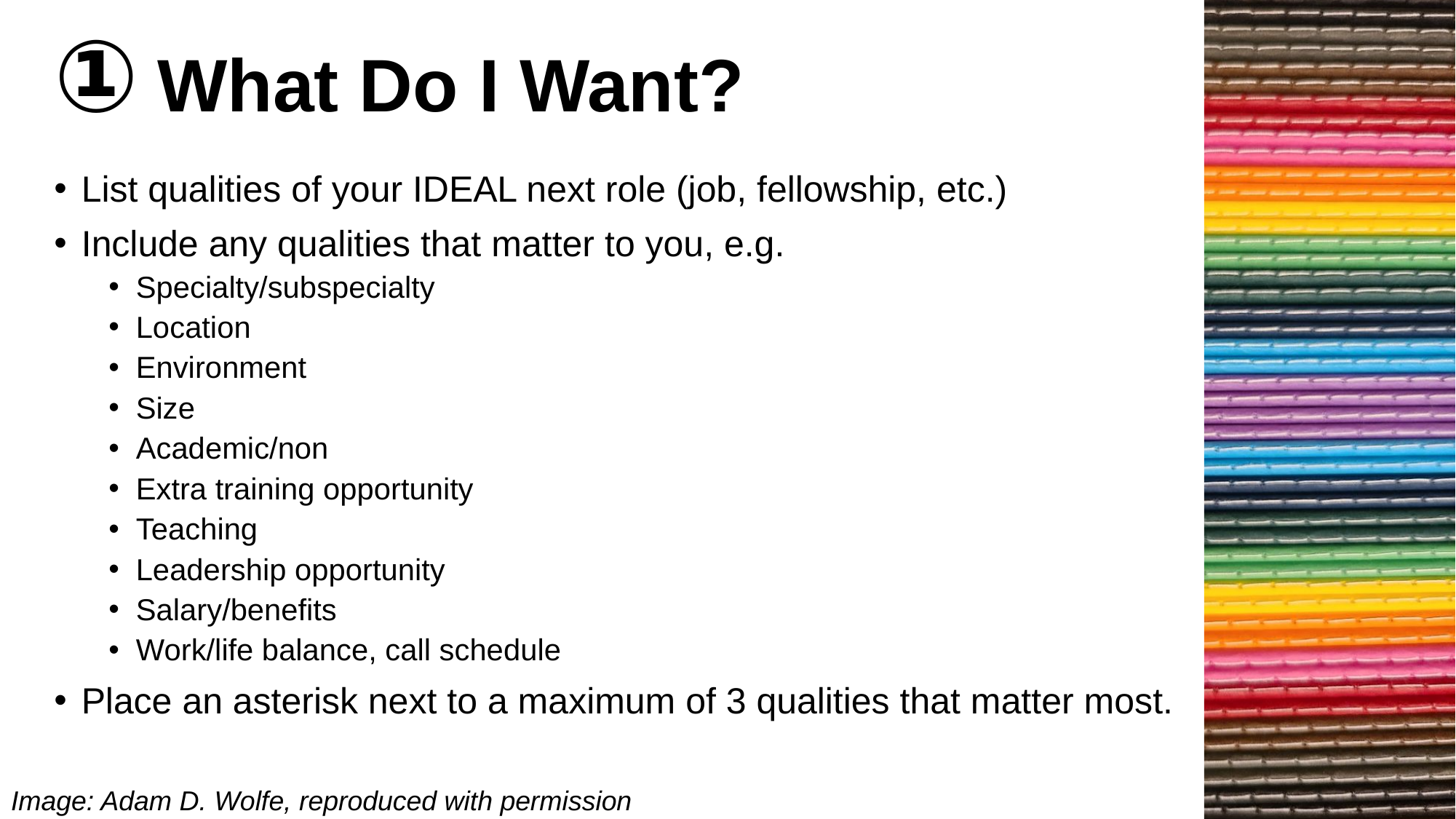

# ① What Do I Want?
List qualities of your IDEAL next role (job, fellowship, etc.)
Include any qualities that matter to you, e.g.
Specialty/subspecialty
Location
Environment
Size
Academic/non
Extra training opportunity
Teaching
Leadership opportunity
Salary/benefits
Work/life balance, call schedule
Place an asterisk next to a maximum of 3 qualities that matter most.
Image: Adam D. Wolfe, reproduced with permission

## Slide 8
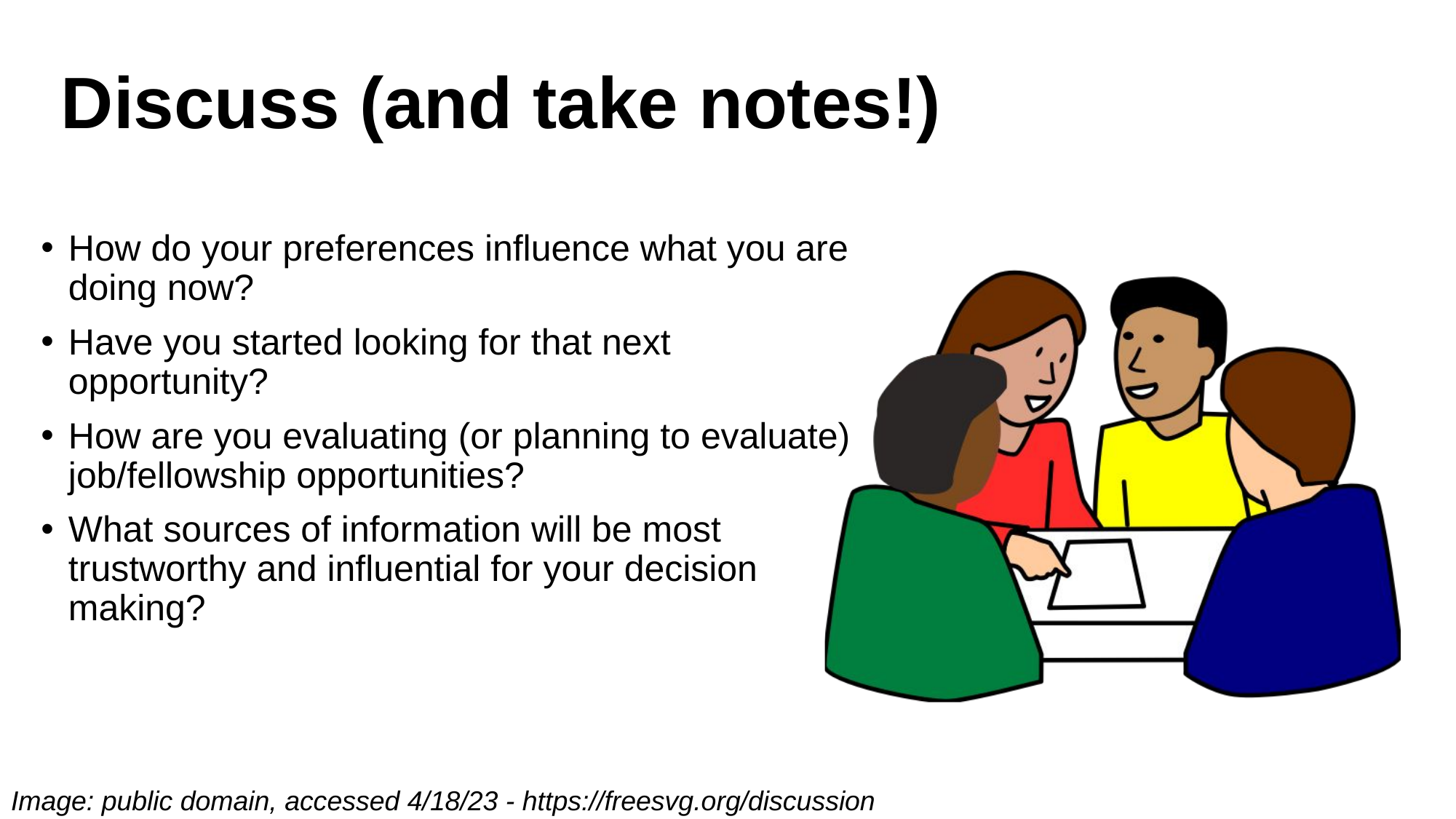

Discuss (and take notes!)
How do your preferences influence what you are doing now?
Have you started looking for that next opportunity?
How are you evaluating (or planning to evaluate) job/fellowship opportunities?
What sources of information will be most trustworthy and influential for your decision making?
Image: public domain, accessed 4/18/23 - https://freesvg.org/discussion

## Slide 9
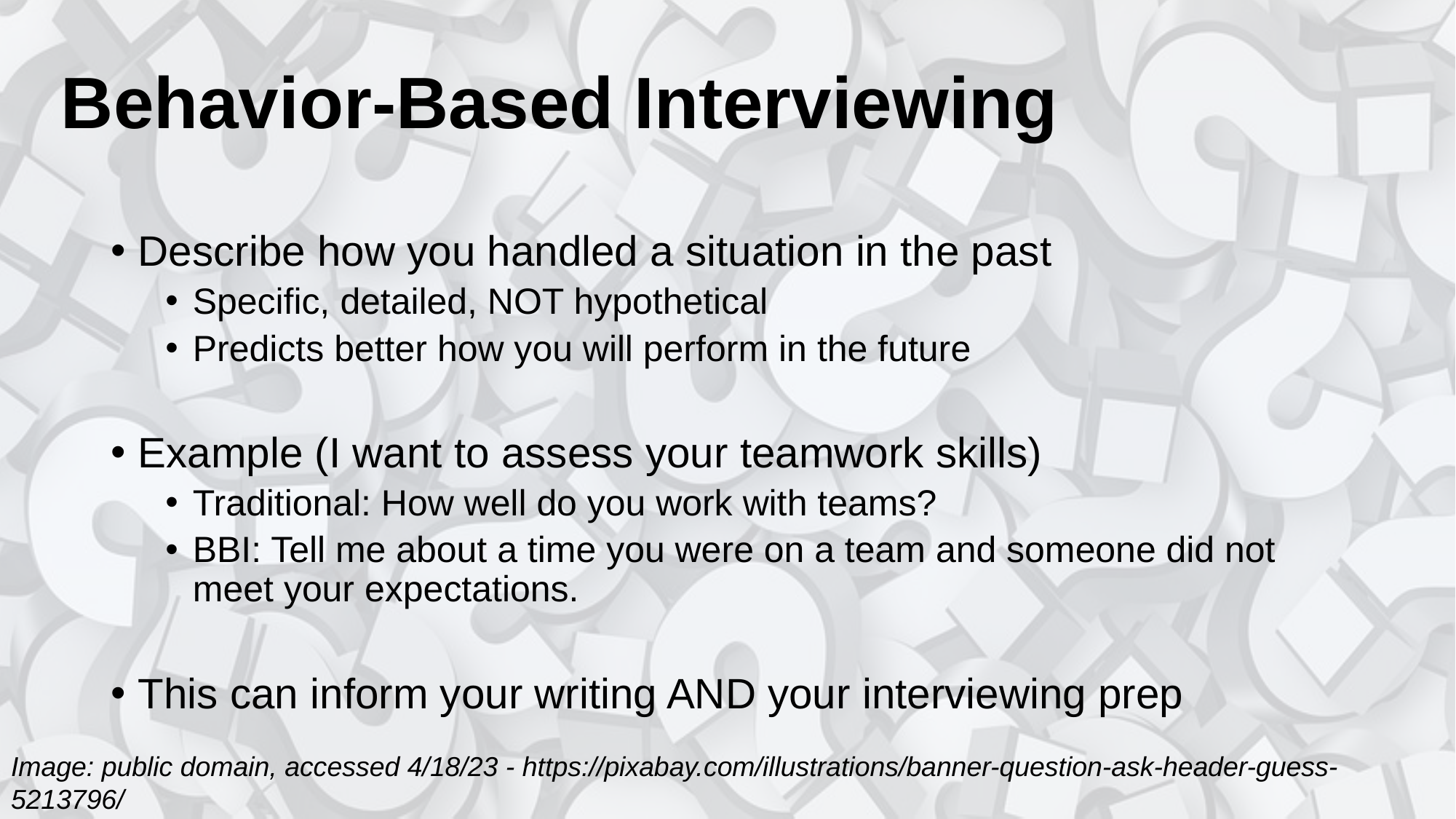

Behavior-Based Interviewing
Describe how you handled a situation in the past
Specific, detailed, NOT hypothetical
Predicts better how you will perform in the future
Example (I want to assess your teamwork skills)
Traditional: How well do you work with teams?
BBI: Tell me about a time you were on a team and someone did not meet your expectations.
This can inform your writing AND your interviewing prep
Image: public domain, accessed 4/18/23 - https://pixabay.com/illustrations/banner-question-ask-header-guess-5213796/

## Slide 10
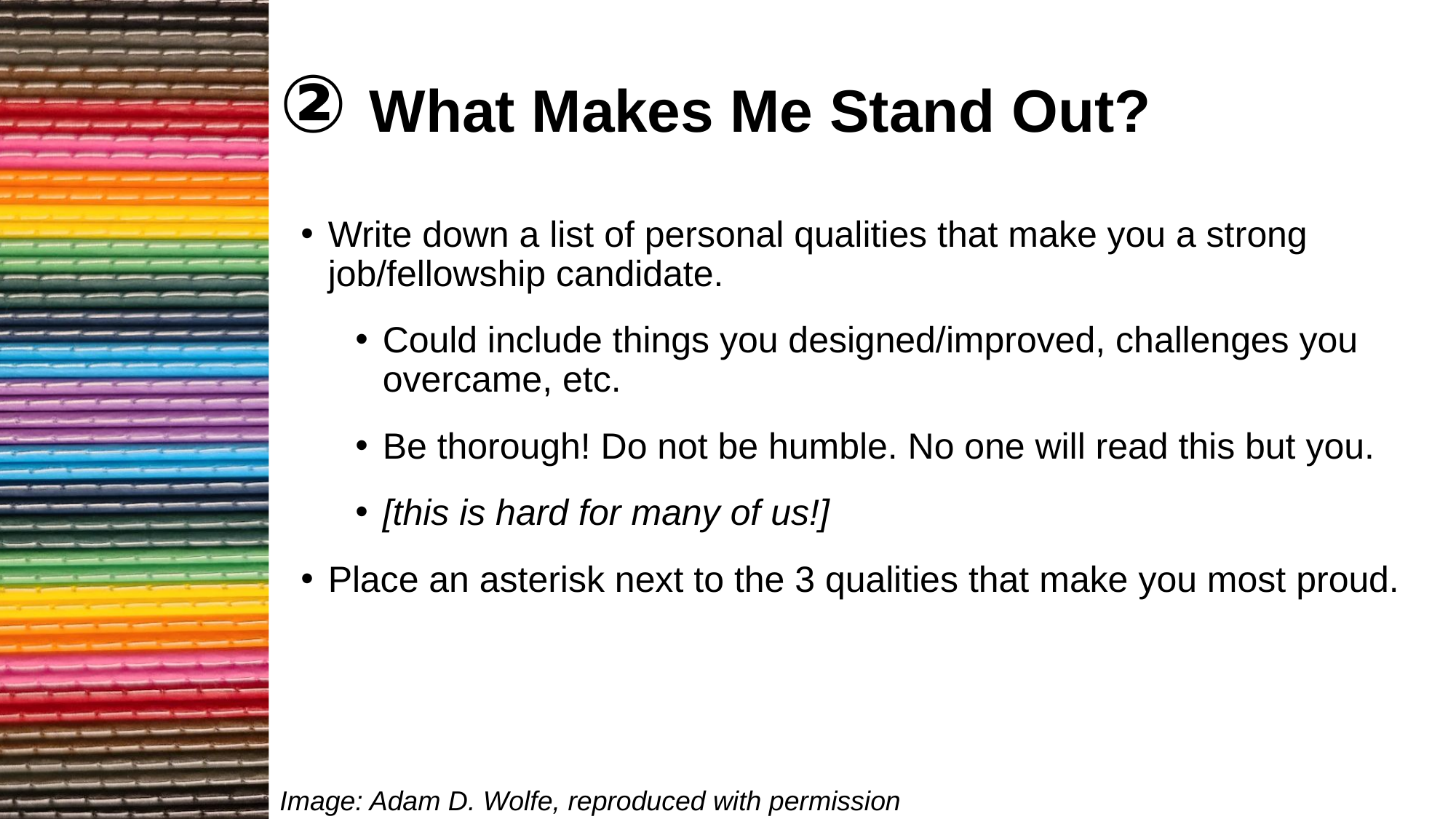

② What Makes Me Stand Out?
Write down a list of personal qualities that make you a strong job/fellowship candidate.
Could include things you designed/improved, challenges you overcame, etc.
Be thorough! Do not be humble. No one will read this but you.
[this is hard for many of us!]
Place an asterisk next to the 3 qualities that make you most proud.
Image: Adam D. Wolfe, reproduced with permission

## Slide 11
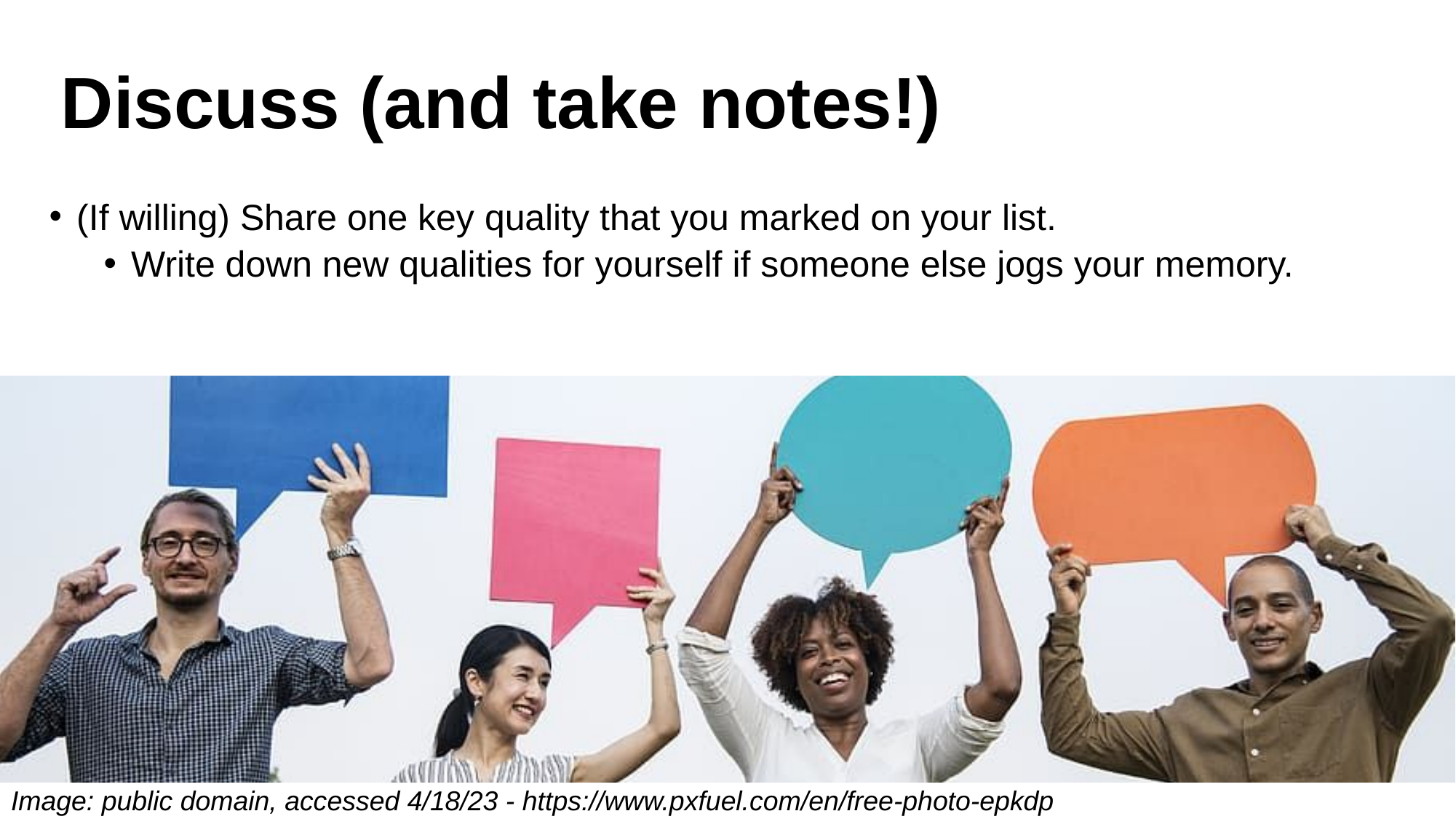

Discuss (and take notes!)
(If willing) Share one key quality that you marked on your list.
Write down new qualities for yourself if someone else jogs your memory.
Image: public domain, accessed 4/18/23 - https://www.pxfuel.com/en/free-photo-epkdp

## Slide 12
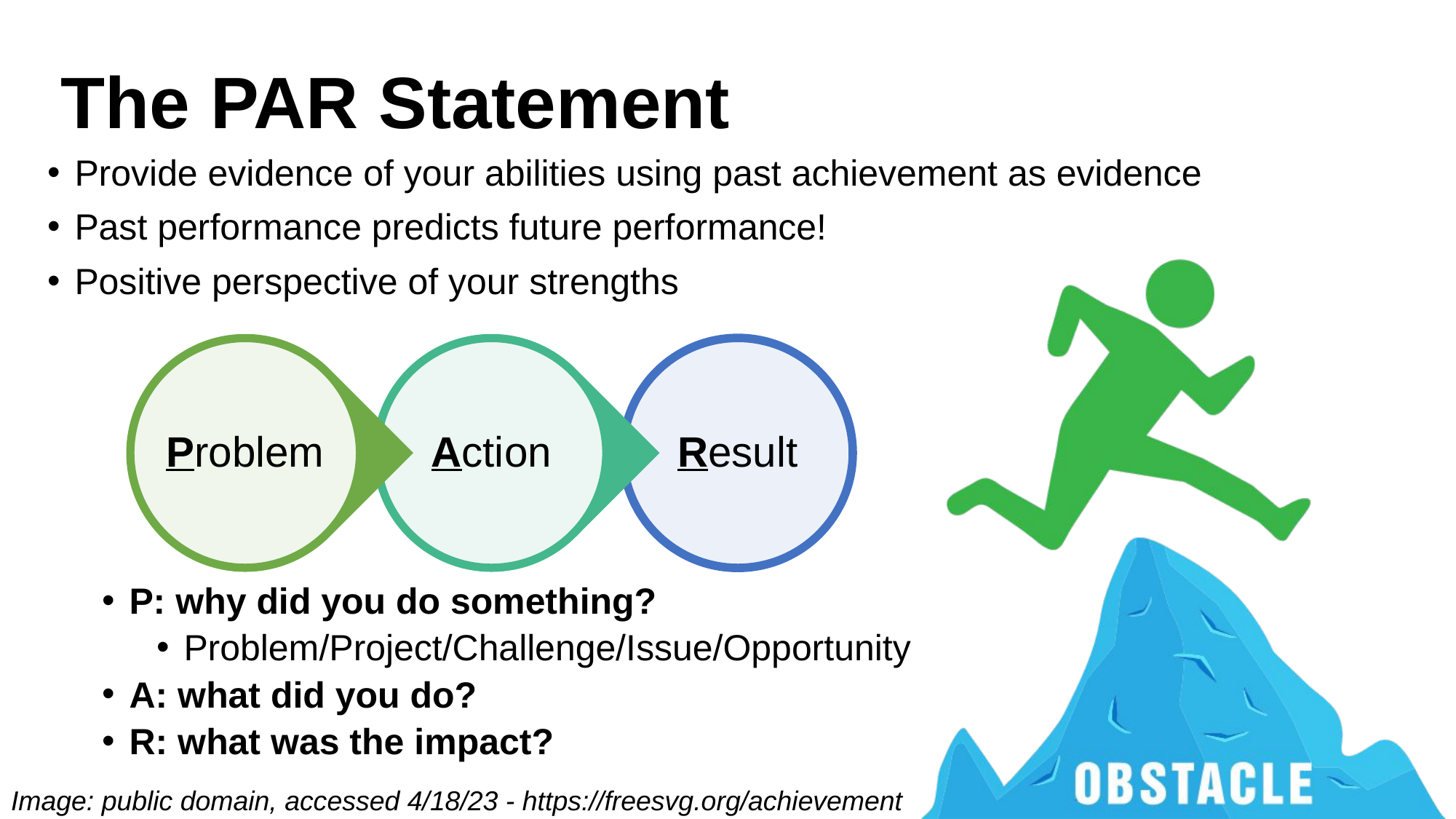

The PAR Statement
Provide evidence of your abilities using past achievement as evidence
Past performance predicts future performance!
Positive perspective of your strengths
P: why did you do something?
Problem/Project/Challenge/Issue/Opportunity
A: what did you do?
R: what was the impact?
Problem
Action
Result
Image: public domain, accessed 4/18/23 - https://freesvg.org/achievement

## Slide 13
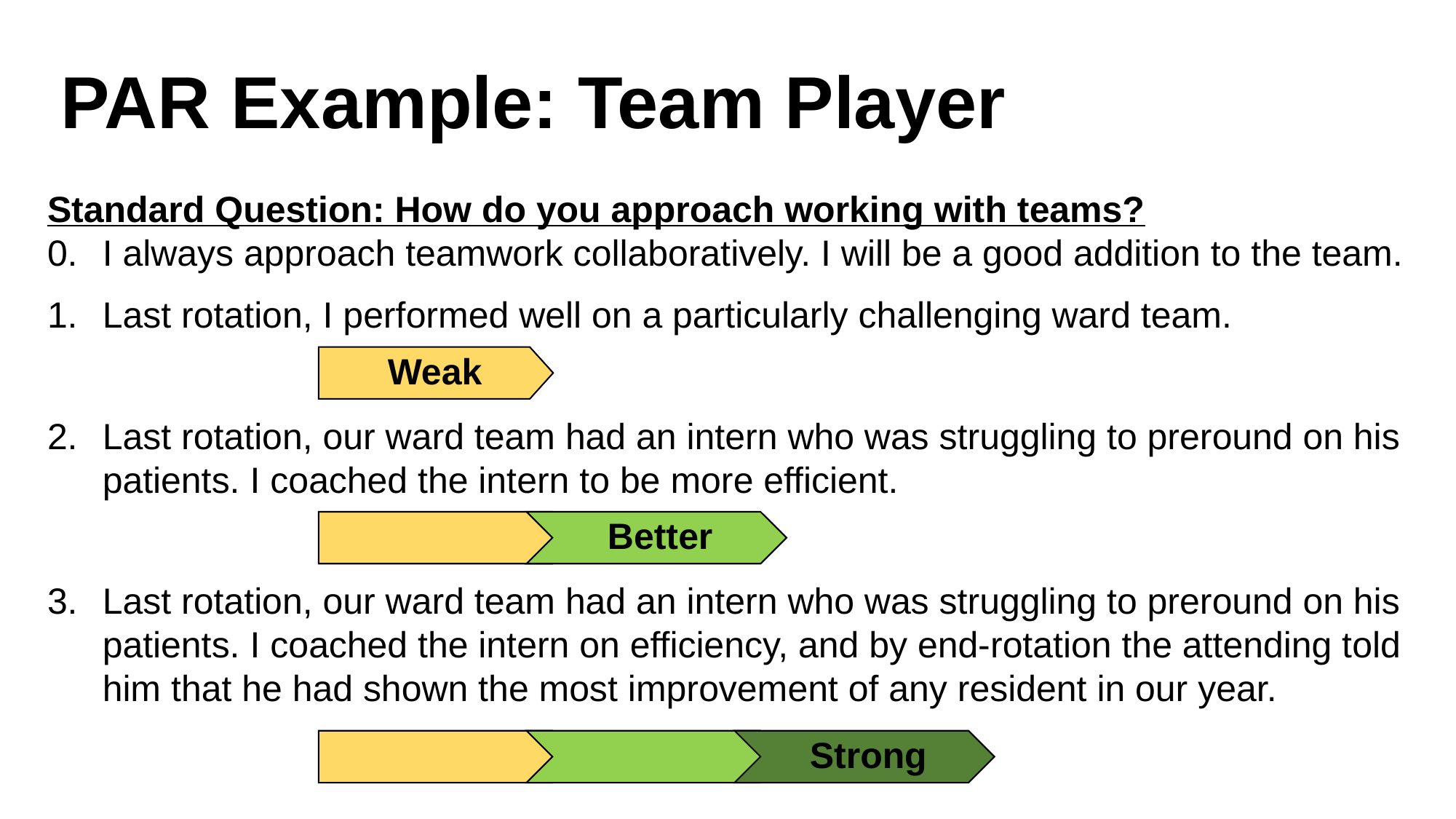

PAR Example: Team Player
Standard Question: How do you approach working with teams?
0.	I always approach teamwork collaboratively. I will be a good addition to the team.
1.	Last rotation, I performed well on a particularly challenging ward team.
2.	Last rotation, our ward team had an intern who was struggling to preround on his patients. I coached the intern to be more efficient.
3.	Last rotation, our ward team had an intern who was struggling to preround on his patients. I coached the intern on efficiency, and by end-rotation the attending told him that he had shown the most improvement of any resident in our year.
Weak
Better
Strong

## Slide 14
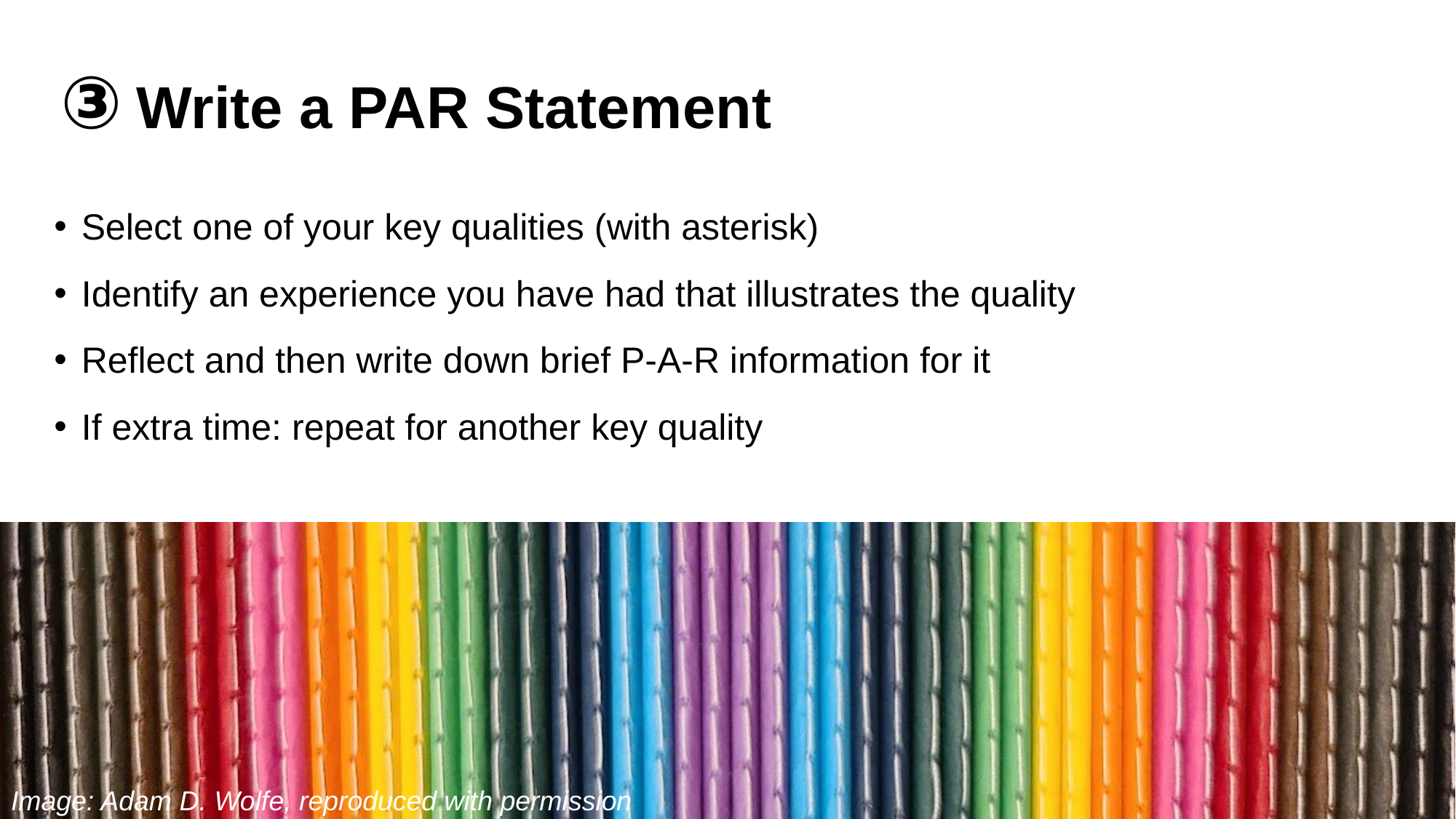

③ Write a PAR Statement
Select one of your key qualities (with asterisk)
Identify an experience you have had that illustrates the quality
Reflect and then write down brief P-A-R information for it
If extra time: repeat for another key quality
Image: Adam D. Wolfe, reproduced with permission

## Slide 15
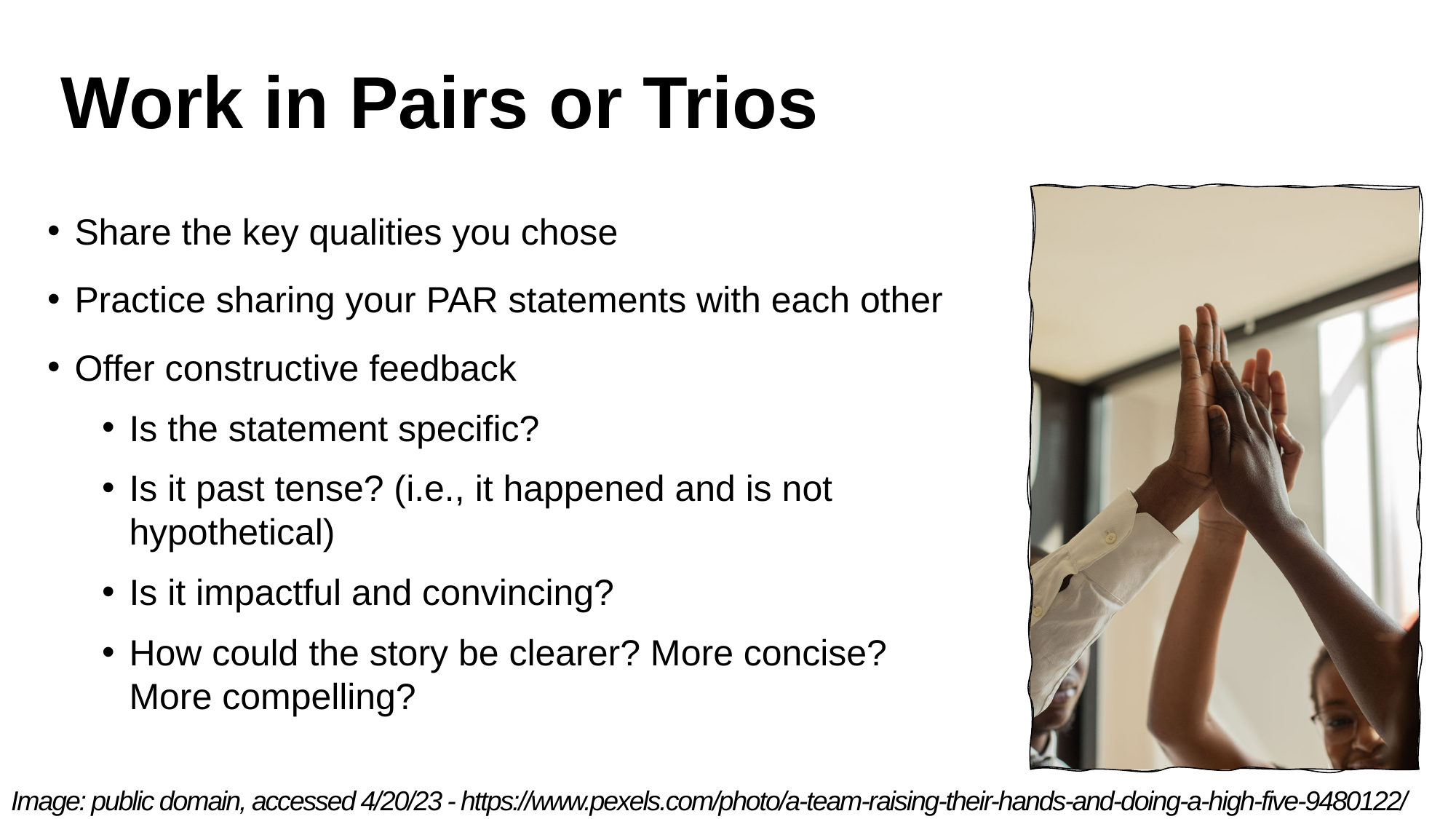

Work in Pairs or Trios
Share the key qualities you chose
Practice sharing your PAR statements with each other
Offer constructive feedback
Is the statement specific?
Is it past tense? (i.e., it happened and is not hypothetical)
Is it impactful and convincing?
How could the story be clearer? More concise? More compelling?
Image: public domain, accessed 4/20/23 - https://www.pexels.com/photo/a-team-raising-their-hands-and-doing-a-high-five-9480122/

## Slide 16
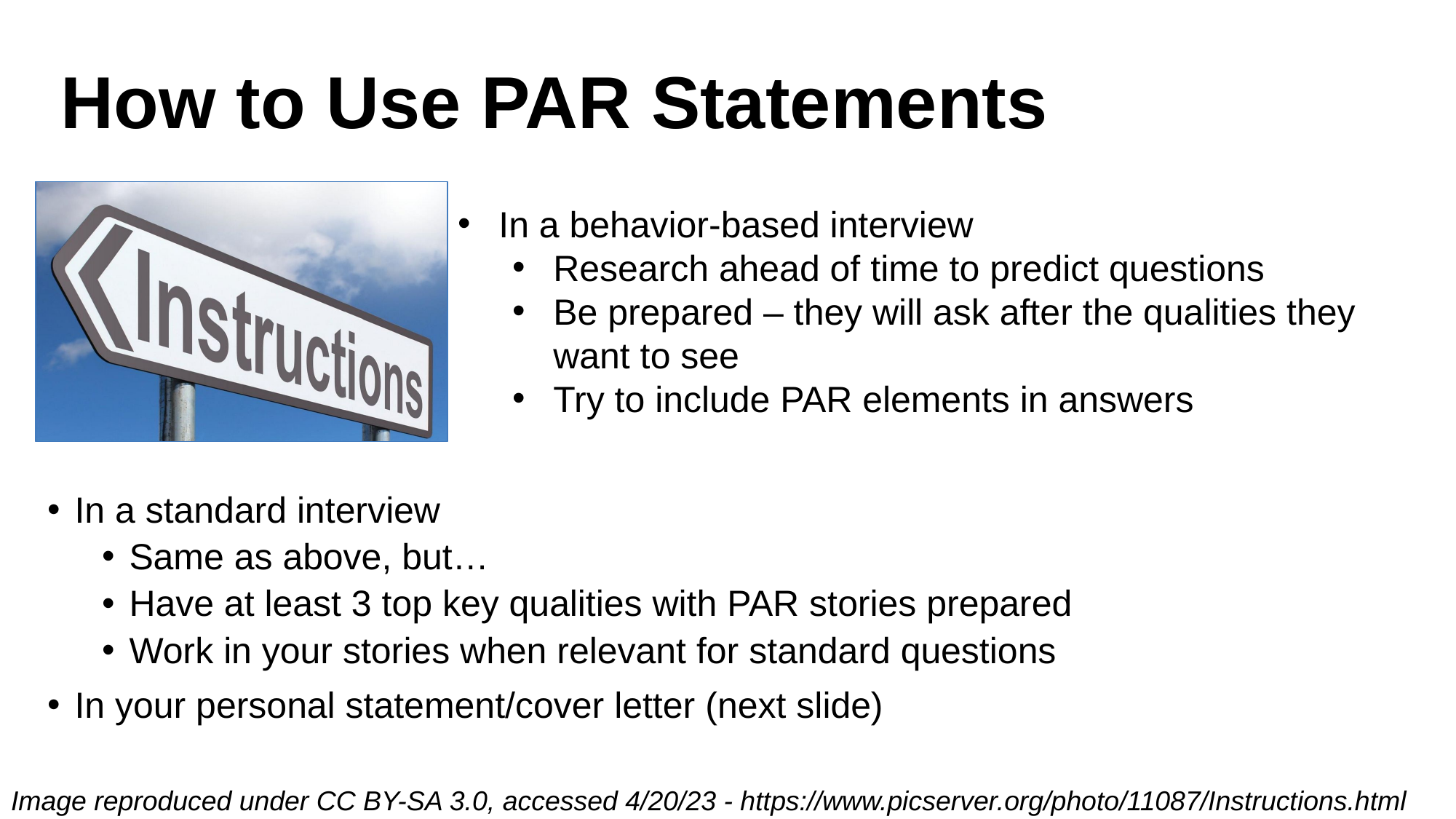

How to Use PAR Statements
In a behavior-based interview
Research ahead of time to predict questions
Be prepared – they will ask after the qualities they want to see
Try to include PAR elements in answers
In a standard interview
Same as above, but…
Have at least 3 top key qualities with PAR stories prepared
Work in your stories when relevant for standard questions
In your personal statement/cover letter (next slide)
Image reproduced under CC BY-SA 3.0, accessed 4/20/23 - https://www.picserver.org/photo/11087/Instructions.html

## Slide 17
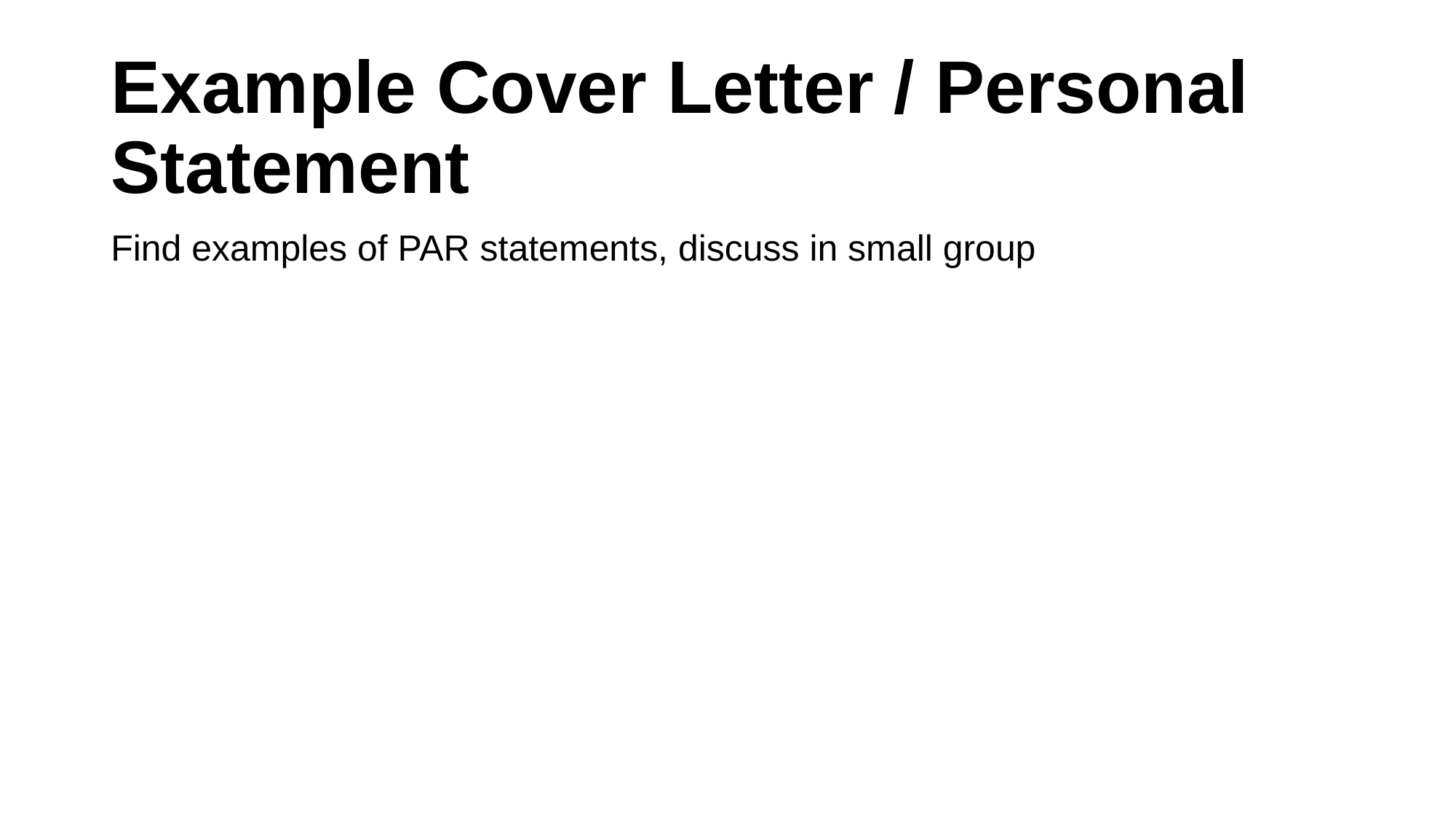

# Example Cover Letter / Personal Statement
Find examples of PAR statements, discuss in small group

## Slide 18
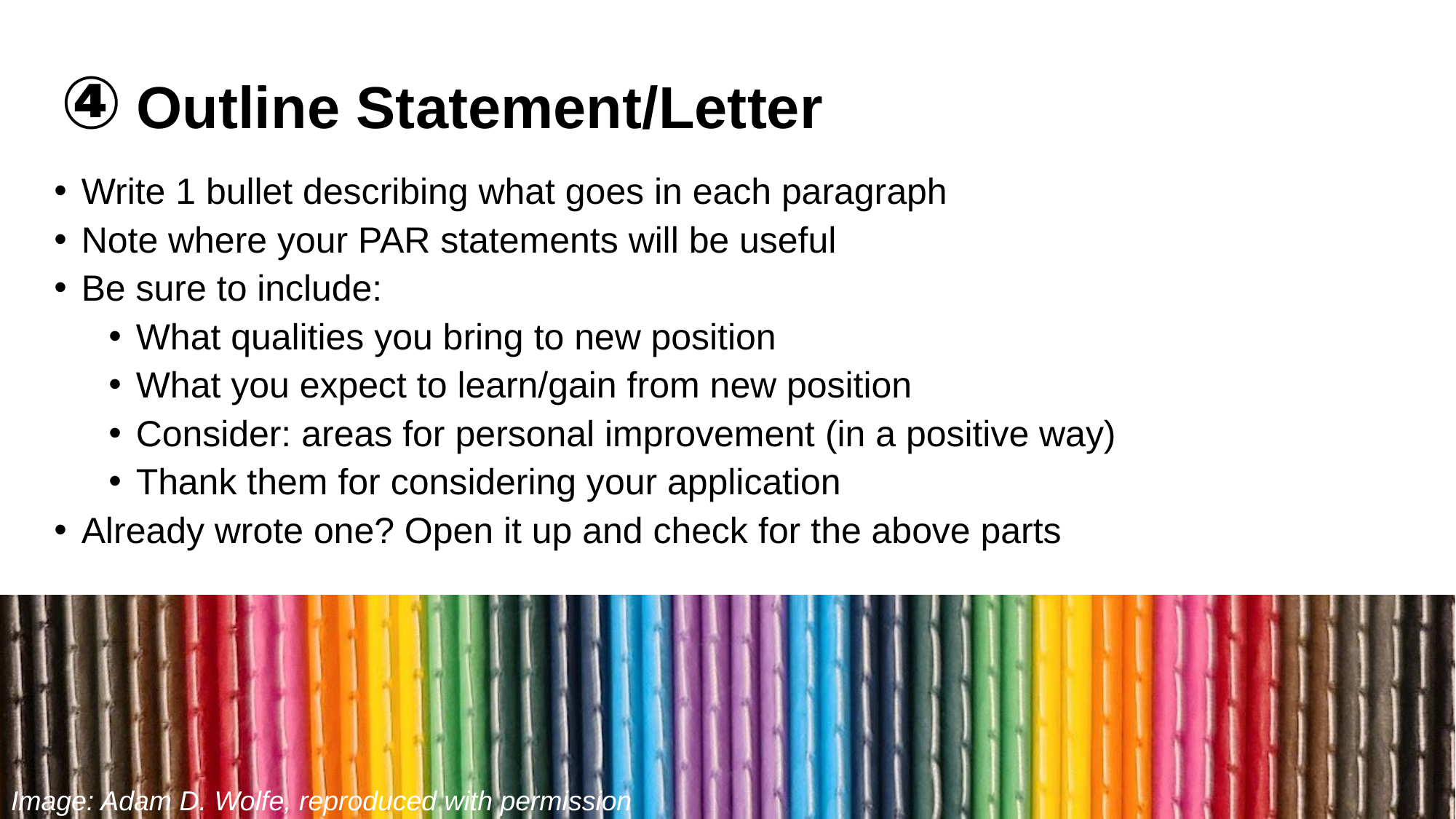

④ Outline Statement/Letter
Write 1 bullet describing what goes in each paragraph
Note where your PAR statements will be useful
Be sure to include:
What qualities you bring to new position
What you expect to learn/gain from new position
Consider: areas for personal improvement (in a positive way)
Thank them for considering your application
Already wrote one? Open it up and check for the above parts
Image: Adam D. Wolfe, reproduced with permission

## Slide 19
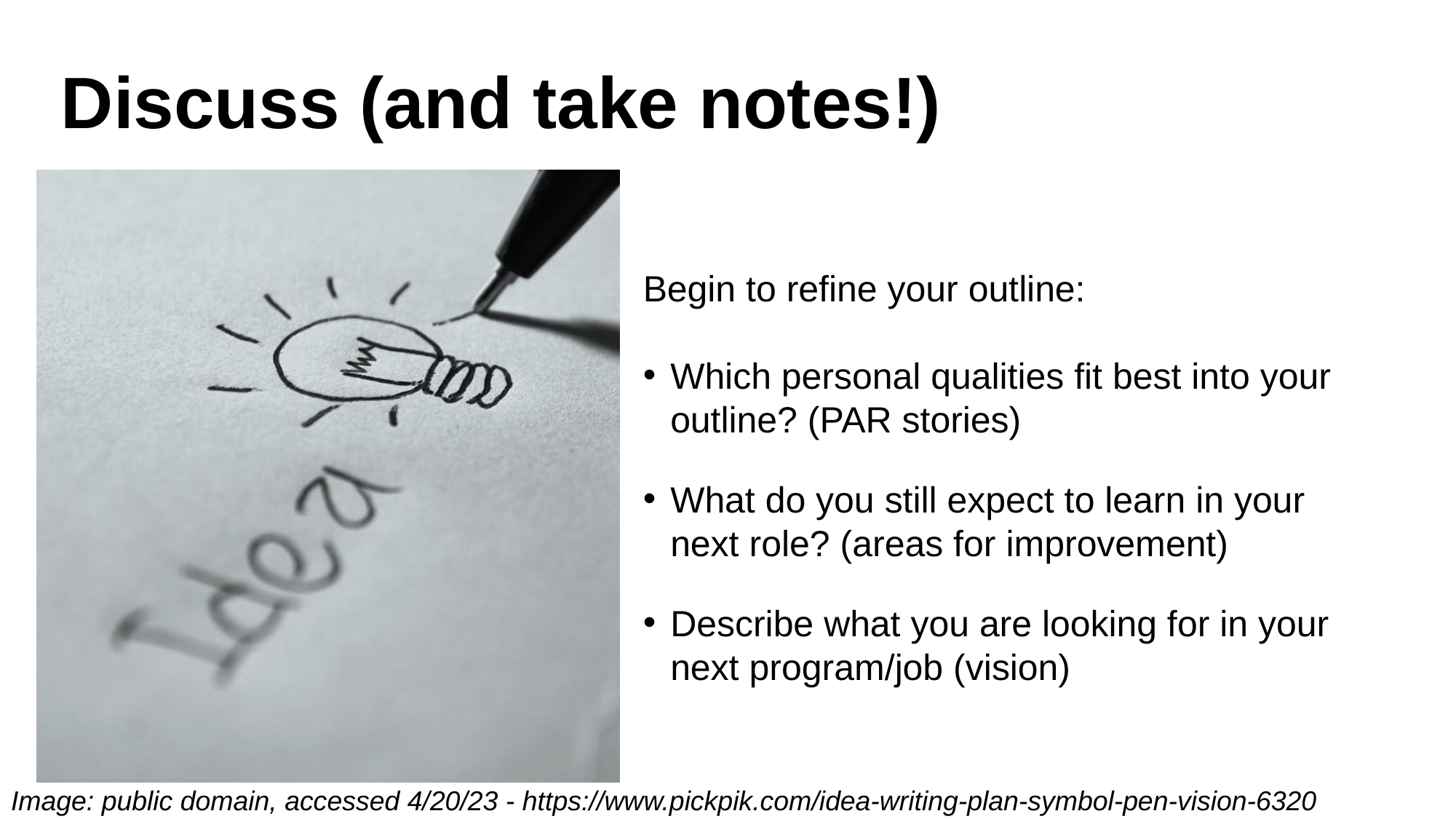

Discuss (and take notes!)
Begin to refine your outline:
Which personal qualities fit best into your outline? (PAR stories)
What do you still expect to learn in your next role? (areas for improvement)
Describe what you are looking for in your next program/job (vision)
Image: public domain, accessed 4/20/23 - https://www.pickpik.com/idea-writing-plan-symbol-pen-vision-6320

## Slide 20
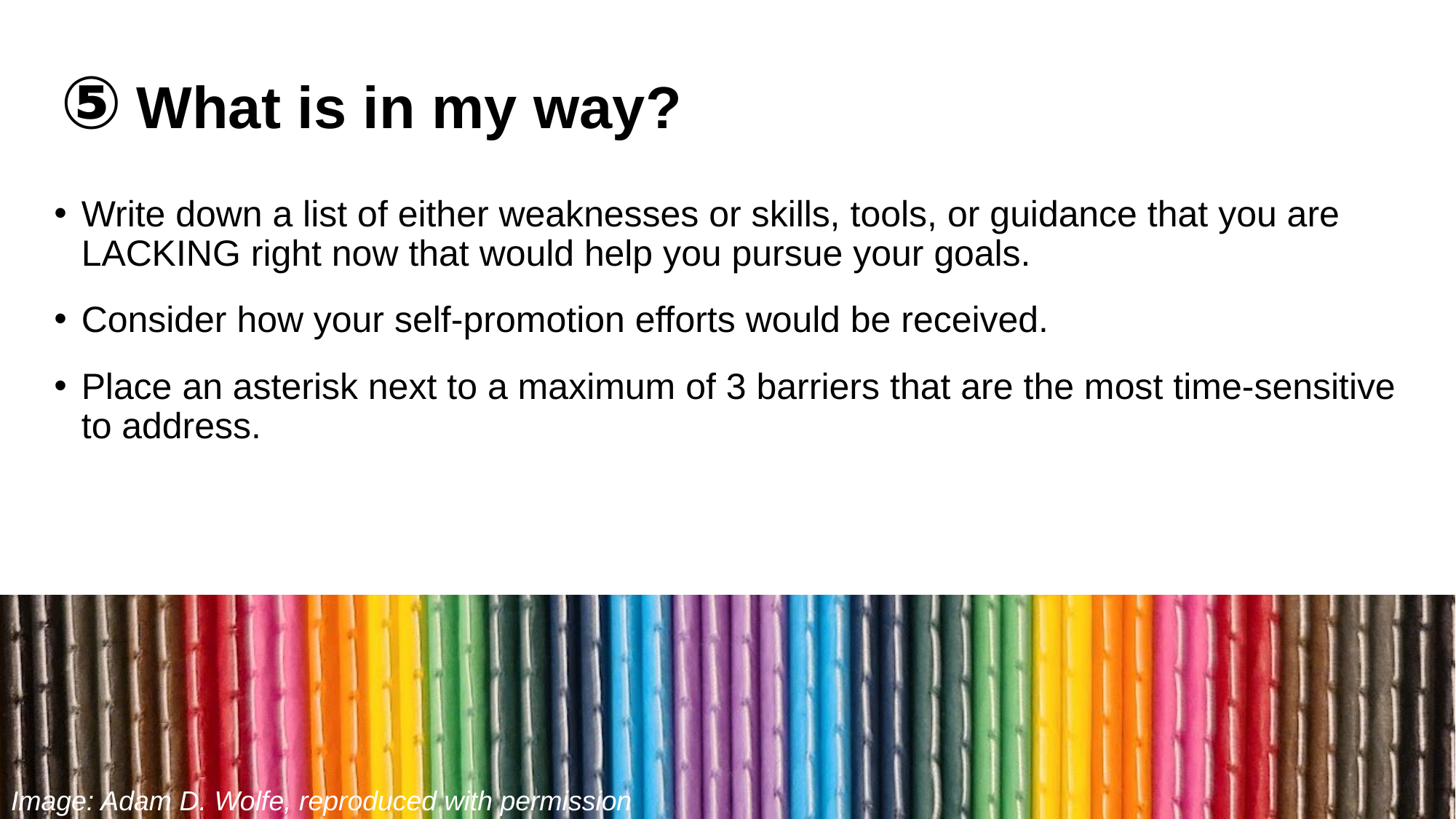

⑤ What is in my way?
Write down a list of either weaknesses or skills, tools, or guidance that you are LACKING right now that would help you pursue your goals.
Consider how your self-promotion efforts would be received.
Place an asterisk next to a maximum of 3 barriers that are the most time-sensitive to address.
Image: Adam D. Wolfe, reproduced with permission

## Slide 21
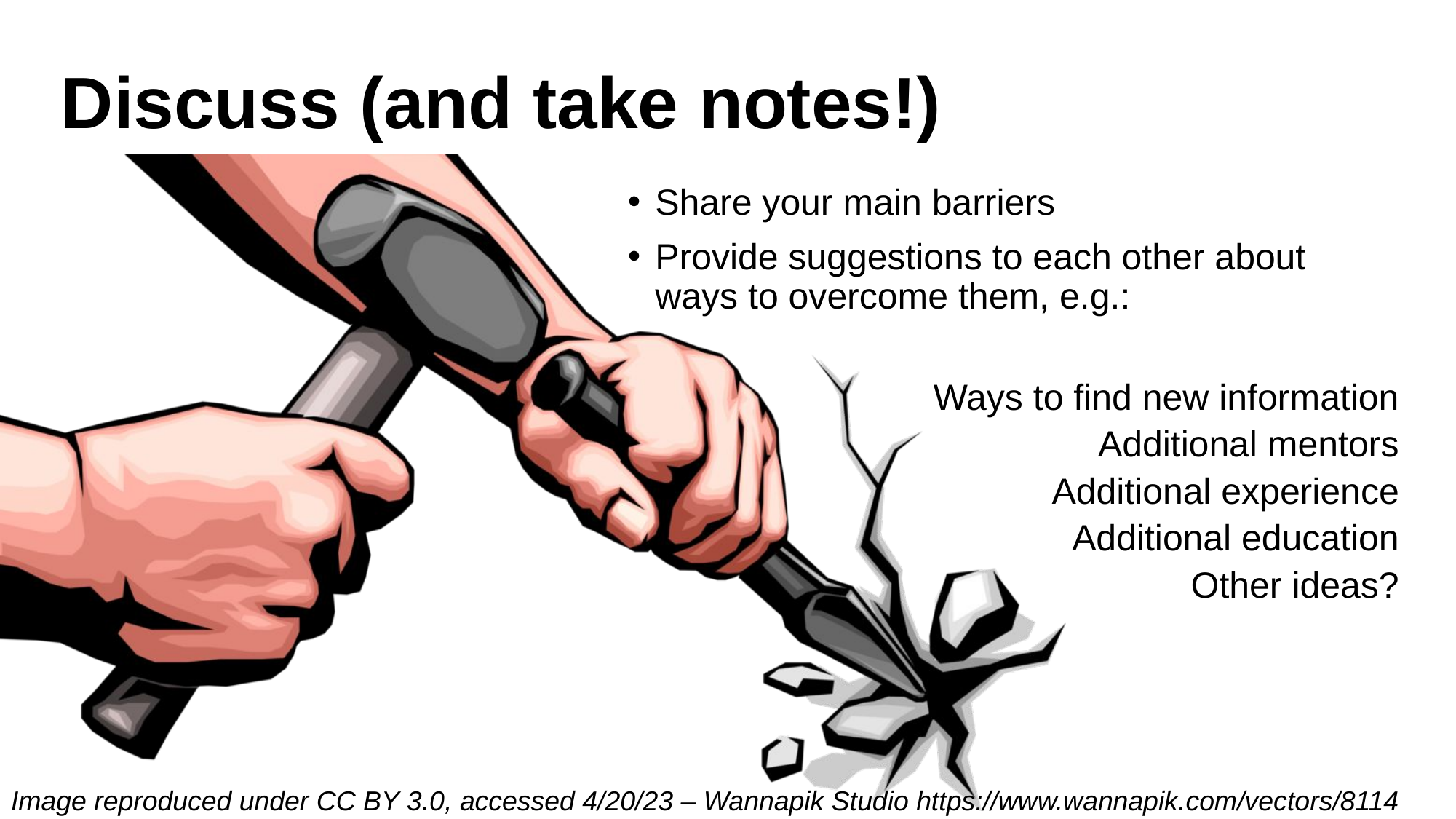

Discuss (and take notes!)
Share your main barriers
Provide suggestions to each other about ways to overcome them, e.g.:
Ways to find new information
Additional mentors
Additional experience
Additional education
Other ideas?
Image reproduced under CC BY 3.0, accessed 4/20/23 – Wannapik Studio https://www.wannapik.com/vectors/8114

## Slide 22
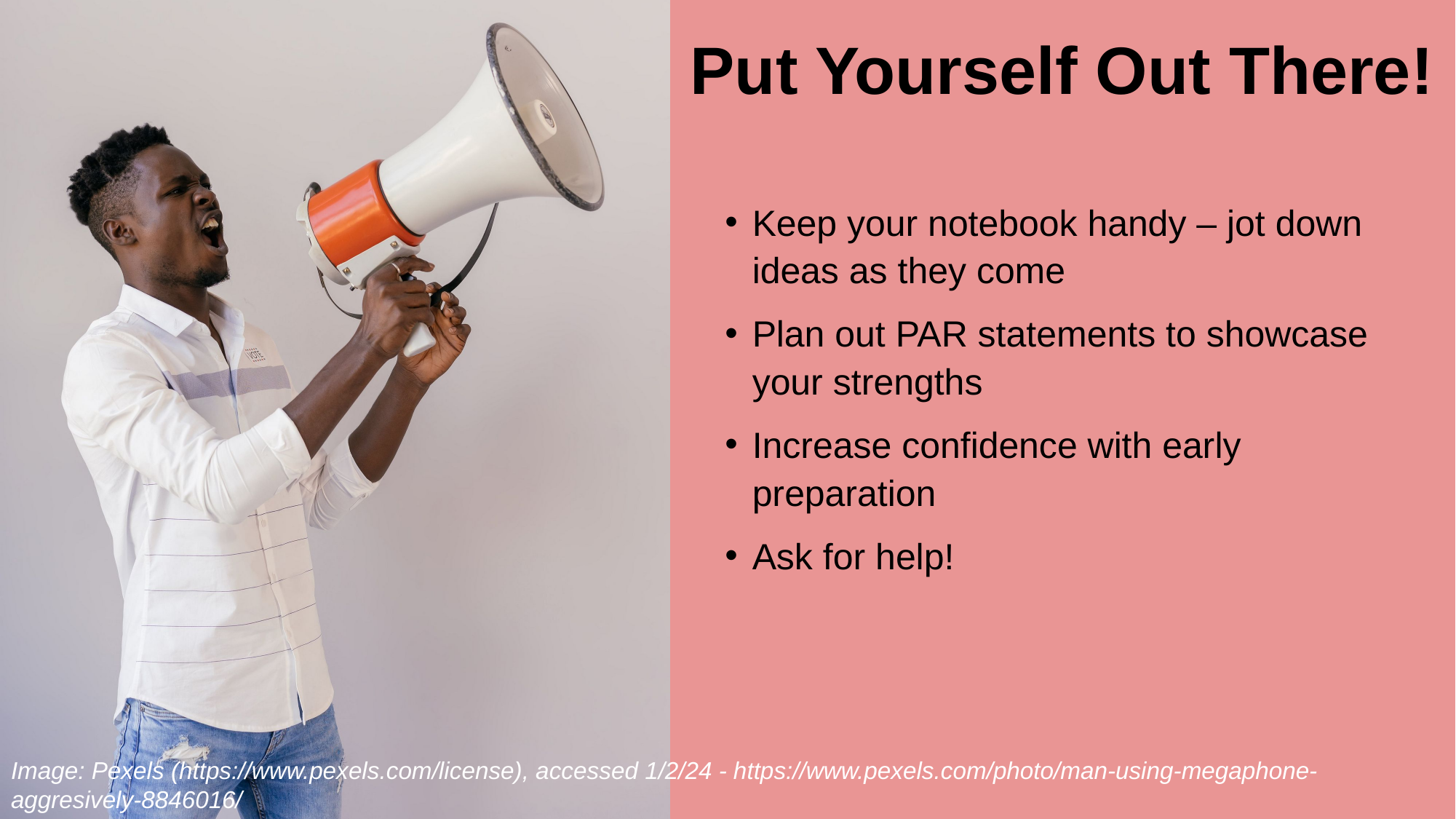

Put Yourself Out There!
Keep your notebook handy – jot down ideas as they come
Plan out PAR statements to showcase your strengths
Increase confidence with early preparation
Ask for help!
Image: Pexels (https://www.pexels.com/license), accessed 1/2/24 - https://www.pexels.com/photo/man-using-megaphone-aggresively-8846016/
